# Functional and genetic evidence that nucleoside transport is highly conserved in *Leishmania* species: Implications for pyrimidine-based chemotherapy

**DOI:** 10.1016/j.ijpddr.2017.04.003

**Published:** 2017-04-20

**Authors:** Khalid J.H. Alzahrani, Juma A.M. Ali, Anthonius A. Eze, Wan Limm Looi, Daniel N.A. Tagoe, Darren J. Creek, Michael P. Barrett, Harry P. de Koning

**Affiliations:** a Institute of Infection, Immunity and Inflammation, College of Medical, Veterinary and Life Sciences, University of Glasgow, Glasgow, United Kingdom; b Department of Clinical Laboratory, College of Applied Medical Sciences, Taif University, Taif, Saudi Arabia; c Al Jabal Al Gharbi University, Gharyan, Libya; d Department of Medical Biochemistry, College of Medicine, University of Nigeria, Enugu Campus, Enugu, Nigeria; e Wellcome Trust Centre for Molecular Parasitology, College of Medical, Veterinary and Life Sciences, University of Glasgow, Glasgow, United Kingdom; f Department of Drug Delivery, Disposition and Dynamics, Monash Institute of Pharmaceutical Sciences, Monash University, Parkville, Victoria, Australia

**Keywords:** *Leishmania*, Pyrimidine metabolism, Uracil transporter, Metabolomics, Nucleoside transporter, 5-fluorouracil, Pyrimidine chemotherapy

## Abstract

*Leishmania* pyrimidine salvage is replete with opportunities for therapeutic intervention with enzyme inhibitors or antimetabolites. Their uptake into cells depends upon specific transporters; therefore it is essential to establish whether various *Leishmania* species possess similar pyrimidine transporters capable of drug uptake. Here, we report a comprehensive characterization of pyrimidine transport in *L. major* and *L. mexicana*. In both species, two transporters for uridine/adenosine were detected, one of which also transported uracil and the antimetabolites 5-fluoruracil (5-FU) and 5F,2′deoxyuridine (5F,2′dUrd), and was designated uridine-uracil transporter 1 (UUT1); the other transporter mediated uptake of adenosine, uridine, 5F,2′dUrd and thymidine and was designated Nucleoside Transporter 1 (NT1). To verify the reported *L. donovani* model of two NT1-like genes encoding uridine/adenosine transporters, and an NT2 gene encoding an inosine transporter, we cloned the corresponding *L. major* and *L. mexicana* genes, expressing each in *T. brucei*. Consistent with the *L. donovani* reports, the NT1-like genes of either species mediated the adenosine-sensitive uptake of [^3^H]-uridine but not of [^3^H]-inosine. Conversely, the NT2-like genes mediated uptake of [^3^H]-inosine but not [^3^H]-uridine. Among pyrimidine antimetabolites tested, 5-FU and 5F,2′dUrd were the most effective antileishmanials; resistance to both analogs was induced in *L. major* and *L. mexicana*. In each case it was found that the resistant cells had lost the transport capacity for the inducing drug. Metabolomics analysis found that the mechanism of action of 5-FU and 5F-2′dUrd was similar in both *Leishmania* species, with major changes in deoxynucleotide metabolism. We conclude that the pyrimidine salvage system is highly conserved in *Leishmania* species - essential information for the development of pyrimidine-based chemotherapy.

## Highlights

• *Leishmania* express 2 adenosine/pyrimidine transporters, 1 oxopurine transporter.
• One of the pyrimidine transporters mediates uracil uptake.
• Some fluorinated pyrimidines are toxic to Leishmania; resistance could be induced.
• The resistant lines lacked capacity to take up fluorinated pyrimidines.
• *Leishmania* metabolism of fluorinated pyrimidines was different from *Trypanosoma*.

## Graphical abstract

**Figure:**
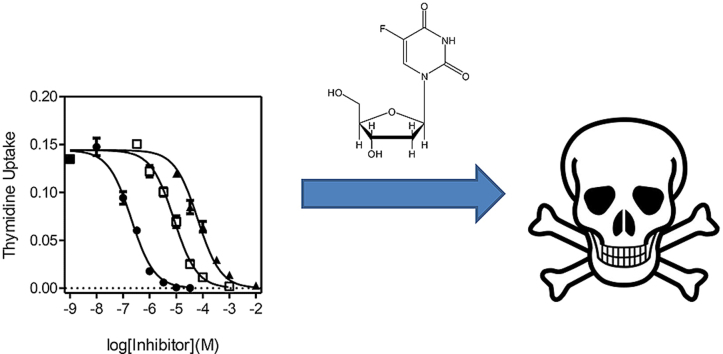

## Nonstandard abbreviations

5-FURes: 5-Fluorouracil resistant cells
5-F2′dURes: 5-Fluoro-2′-deoxyuridine resistant cells
5-FU: 5-Fluorouracil
5F-2′dUrd: 5-Fluoro-2′-deoxyuridine
5F-Urd: 5-Fluorouridine
5F-2′dCtd: 5-Fluoro-2′-deoxycytidine

## 1 Introduction

Leishmaniasis is a neglected tropical disease caused by at least 21 species of obligate intracellular parasites of the genus *Leishmania*, and is transmitted by more than 30 species of female sand-flies from the genera *Phlebotomus* (Old World) or *Lutzomyia* (New World) (Rodrigues et al., 2014). The disease remains a major cause of morbidity and mortality worldwide that has been classified into three main categories on the basis of clinical symptoms: visceral leishmaniasis (VL), cutaneous leishmaniasis (CL) and mucocutaneous leishmaniasis (MCL) (Alvar et al., 2012). Due, in part, to the fact that leishmaniasis with various clinical manifestations is caused by different species, treatment of the disease is still complicated, and often unsatisfactory (Croft and Olliaro, 2011, Sundar and Chakravarty, 2013). Nucleotide metabolism provides many promising therapeutic targets due to the fact that protozoan parasites are unable to synthesize the purine ring *de novo* and rely solely on salvage mechanisms for these important nutrients (De Koning et al., 2005). Nonetheless, purine analog-based chemotherapy has not emerged against kinetoplastid parasites due to redundancy of the interconversion pathways, making inhibition of single enzymes often ineffective (Lüscher et al., 2007a, Lüscher et al., 2013, Berg et al., 2010a). For the major protozoan pathogens most of the purine nucleoside and nucleobase transporters – which also sometimes exhibit a secondary transport activity for pyrimidines – have been cloned, and all of these transporters belonged to the Equilibrative Nucleoside Transporter (ENT) family (e. g. Vasudevan et al., 1998, Chiang et al., 1999, Burchmore et al., 2003, Sanchez et al., 2004, De Koning et al., 2005, Quashie et al., 2008). In contrast to purines, kinetoplastid parasites are known to possess both salvage and biosynthesis routes for pyrimidines (Wilson et al., 2012, Ali et al., 2013a, Ali et al., 2013b). It has recently been demonstrated that although both functions are important for infection, neither function is absolutely essential (Wilson et al., 2012, Ali et al., 2013b).

Although no single *Leishmania* purine or pyrimidine transporter can be considered essential (Ortiz et al., 2007, Wilson et al., 2012), they are vital links in the delivery of any nucleoside-based chemotherapy to these parasites. The first purine transporter genes to be identified from any parasite were LdNT1 (Vasudevan et al., 1998) and LdNT2, which were cloned from *L. donovani* (Carter et al., 2000). NT1 mediates the uptake of adenosine and the pyrimidine nucleosides uridine and thymidine, whereas NT2 recognizes the 6-oxopurine nucleosides inosine, guanosine, and xanthosine (Carter et al., 2001, Boitz et al., 2012). NT1 and NT2 also serve as the primary conduits for uptake of the antileishmanial adenosine analog tubercidin (7-deazaadenosine) and the antileishmanial inosine analog formycin B, respectively (Vasudevan et al., 1998, Carter et al., 2000), but the pharmacological exploitation of these transporters has not been investigated further. In addition, two purine nucleobase transporter genes have been described in *L. major*, encoding the broad specificity nucleobase transporter LmajNT3 active in promastigotes (Sanchez et al., 2004), and the acid-activated LmajNT4 that is presumed to be mainly functional in the intracellular amastigotes (Ortiz et al., 2009). It is assumed that LmajNT3 and LmajNT4 correspond to the previously characterized nucleobase transport activities in *L. major* promastigotes and *L. mexicana* amastigotes, respectively (Al-Salabi et al., 2003, Al-Salabi and De Koning, 2005), and that there are at a minimum some differences between nucleoside/nucleobase transport in the promastigote and amastigote stages (Ghosh and Mukherjee, 2000, De Koning et al., 2005). Finally, a uracil-specific transporter designated LmU1 was characterized in *L. major* promastigotes (Papageorgiou et al., 2005), but unlike the NT1-4 nucleoside and purine nucleobase transporters, which are members of the Equilibrative Nucleoside Transporter (ENT) family, the gene encoding this transporter is unknown and believed to be of a different gene family (De Koning, 2007).

There remain many caveats to the pharmacological exploitation of the purine and/or pyrimidine salvage pathways for antileishmanial chemotherapy, including whether there are significant differences in nucleoside transport activities between the various *Leishmania* species, what antimetabolites might be transported by *Leishmania* nucleoside transporters, or what metabolic activation steps might follow the uptake of pyrimidine antimetabolites. In this study we address some of these issues and find that (1) nucleoside transport is highly similar in multiple *Leishmania* species; (2) that the substrate binding of the LmajNT1 transporter depends on interactions with the 2-keto and N3 positions of the pyrimidine ring and the 3′ and 5′ hydroxyl groups of the ribose moiety; and that (3) the antimetabolite 5-fluoro-2′-deoxyuridine (5F-2′dUrd) is principally converted to 5F-dUMP, by thymidine kinase, causing the inhibition of thymidylate synthase and the consequent disruption of deoxynucleotide metabolism; 5-fluorouracil is first converted to 5F-2′dUrd and thence to 5F-dUMP.

## 2 Materials and methods

### 2.1 Kinetoplastid strains and cultures

Promastigotes of *L. mexicana* (MNY/BZ/62/M379 strain) and *L. major* (Friedlin strain) were grown in HOMEM medium (Gibco, Paisley, UK) (pH 7.4) supplemented with 10% fetal bovine serum (FBS) (Gibco) 1% Penicillin/Streptomycin antibiotic (Gibco) at 25 °C as described (Al-Salabi et al., 2003). The *T. b. brucei* strain B48 (Bridges et al., 2007) was used throughout as the expression system for *Leishmania* transporters, and maintained exactly as described previously in HMI-9 medium with 10% FBS (Gibco) under a 5% CO_2_ atmosphere at 37 °C (Vodnala et al., 2013). This strain is derived from a Lister 427 clone from which the aminopurine transporter *TbAT1* has been deleted (Matovu et al., 2003) and was further adapted to high levels of pentamidine, causing it to additionally lose the High Affinity Pentamidine Transporter (HAPT1) activity, encoded by the gene *TbAQP2* (Munday et al., 2014).

### 2.2 Plasmid construction and transfection

Plasmid construction and transfection was performed according to Munday et al. (2013). The nucleoside transporter genes were isolated from *L. mexicana* and *L. major*. The sequences of these genes displayed a high degree of similarity to the known *L. donovani* nucleoside transporter genes (LdNT1.1, LdNT1.2 and LdNT2; Table S1). Since we could not differentiate between NT1.1 and NT1.2 genes in *L. major* and *L. mexicana* because both genes are highly similar to both LdNT1.1, LdNT1.2, we designated these genes as NT1A and NT1B (Table S1). The primers used in this study (Table S2) were designed to flank the gene of interest, one complementary to the sequence upstream of the 5′ end and the other complementary to the sequence downstream of the 3′ end. All of the nucleoside transporters genes were PCR-amplified from genomic DNA of each strain using the high-fidelity proof-reading polymerase Phusion (New England Biolabs) and cloned into the pGEMTeasy (Promega) vector prior to Sanger sequencing (Source BioScience, Glasgow, UK). For each gene, six independent clones were sequenced and verified as correct. After confirming the identity of each gene, the nucleoside transporter genes (LmajNT1A, LmajNT1B, LmajNT2, LmexNT1A, LmexNT1B, and LmexNT2) were ligated into the expression vector pHD1336 (Biebinger et al., 1997) and then were linearized with *Not*I digestion. All genes were verified by Sanger sequencing, prior to transfection into *T. b. brucei* clone B48. B48 parasites (1 × 10^7^ cells) were washed into Human T Cell buffer for transfection using the desired cassette with an Amaxa Nucleofector using program X-001. Cells were transferred into pre-warmed HMI-9 medium and allowed to recover for 8–16 h at 37 °C and 5% CO_2_. Following recovery, transfectants were grown and cloned in selective medium containing 5 μg ml^−1^ blasticidin S using limiting dilution.

### 2.3 Quantitative real-time PCR (qRT-PCR)

The experiment was performed exactly as described previously (Ali et al., 2013b). Primers for qRT-PCR were designed using Primer3^®^ (Table S3). RNA isolated from *T. b. brucei* B48 strains and *Leishmania* species was quantified using a NanoDrop device; 2 μg of RNA was diluted in RNase-free water to a total volume of 25 μl 200 ng of RNA from each generated and control cell line, were used for the production of complementary DNA (cDNA) using a Reverse-Transcriptase (RT) kit (Primerdesign, UK). For each sample the cDNA was diluted with RNase free water to 20 ng/μl for qRT-PCR. Amplification of cDNA was performed in a 7500 Real Time PCR System (G-STORM, Thermo Scientific). The dissociation curve was used to ensure the amplification of only one product; samples without RT or cDNA were used as controls. The constitutively expressed gene GPI8 was used as an internal control (Wilson et al., 2012). The ΔΔCT method was used for relative quantification (RQ) using *T. b. b.* B48 cells in HMI-9 as a calibrator for the nucleoside transporter genes expressed in *T. b. b.* B48, and using *L. mexicana* promastigotes as a calibrator for the expression level of nucleoside transporters genes in *L. mexicana* amastigotes. Data were analyzed using Applied Biosystems 7500 SDS Real-Time PCR systems software.

### 2.4 Drug sensitivity assays

Sensitivity assays of *Leishmania* strains to various drugs using the viability dye resazurin (Alamar Blue) (Sigma-Aldrich) were performed using a protocol adapted from Räz et al. (1997), as described (Al-Salabi et al., 2003, Gould et al., 2008). Pentamidine and diminazene were used as non-nucleoside controls and were obtained from Sigma-Aldrich, as were many purines, pyrimidines, and analogs, with the exceptions of 2-thiouridine and 4-thiouridine (TriLink BioTechnologies, San Diego, CA); 5′-deoxyuridine, and 2′-3′-dideoxyuridine (Carbosynth, Compton, UK); 5-fluoro-2′-deoxyuridine (Fluka); and 2-thiouracil (ICN Biomedicals, Cambridge, UK). A preliminary promastigote culture was diluted to a density of 2 × 10^6^ cells/ml, of which 100 μl was added to wells of 96-well plates pre-loaded with 100 μl of doubling dilutions of test compounds, resulting in a final density of 1 × 10^6^ cells/ml; the dilutions were over 2 rows of the plate (23 concentrations), with the last well containing only medium and serving as the no-drug control. The plates were incubated at 25 °C for 72 h before adding the Alamar Blue dye (20 μl of 12.5 mg resazurin sodium salt (Sigma) in 100 ml phosphate buffered saline (PBS; pH 7.4)). Since *Leishmania* parasites metabolize the Alamar Blue dye slower than trypanosomes (Gould et al., 2008), the cells were incubated with the dye for a further period of 48 h before measuring the fluorescence, using a FLUOstar Optima fluorimeter (BMG Labtech) at wavelengths of 544 nm for excitation and 620 nm for emission. 50% effective concentrations (EC_50_) were calculated using the equation for a sigmoidal curve with variable slope using Prism 5.0 (GraphPad software Inc, California, USA) software; extrapolation of incomplete curves was used when >50% inhibition was achieved, using the minimum fluorescence in the curve with the control drug (pentamidine) as the sole constraint. Each experiment was performed independently at least 4 times; statistical significance was determined using Student's unpaired *t*-test.

### 2.5 Transport assays

Using the standard uptake technique as described for *T. brucei* and *Leishmania* species (Wallace et al., 2002, Al-Salabi and De Koning, 2005, Gudin et al., 2006), the dose- or time-dependent uptake of radiolabeled permeants was investigated. The following radiolabels and specific activities were used: [^3^H]-thymidine (Perkin Elmer; 56.6 Ci/mmol); [^3^H]-adenosine (American Radiolabeled Chemicals UK; 40 Ci/mmol); [^3^H]-uridine (American Radiolabeled Chemicals UK; 30 Ci/mmol); [^3^H]-uracil (Perkin Elmer; 24.8 Ci/mmol); [^3^H]-inosine (American Radiolabeled Chemicals UK; 20 Ci/mmol); [^3^H]-5-fluorouracil (Moravek; 20 Ci/mmol).

Briefly, cells in the mid-to-late logarithmic stage of growth were harvested by centrifugation for 10 min at 1500×*g*. The cells were washed twice with transport assay buffer (AB: 33 mM HEPES, 98 mM NaCl, 4.6 mM KCl, 0.55 mM CaCl_2_, 0.07 mM MgSO_4_, 5.8 mM NaH_2_PO_4_, 0.3 mM MgCl_2_, 23 mM NaHCO_3_, 14 mM glucose, pH 7.3), resuspended at a density of 10^8^ cells ml^−1^ in AB, and left for 20–30 min at room temperature to recover from centrifugation stress. One hundred microliters of cell suspension was incubated at ambient temperature for a predetermined time with 100 μl radiolabeled test compound, in the presence or absence of unlabeled substrate or other competitive inhibitors. The incubation was terminated by the addition of ice-cold stop solution (AB containing saturating levels, usually 10 mM, of unlabeled permeant) and centrifugation through oil for 1 min at 13,000 × *g*. Trapped extracellular radioactivity was determined as the amount of radiolabel associated with the cell pellet in the presence of 1 mM permeant (i.e. saturation of all high affinity transport activities), and subtracted. Radioactivity was determined by liquid scintillation counting in a Beckman LS6000 TA scintillation counter. Saturation data, inhibition data, and time courses were plotted to equations for linear or non-linear regression (hyperbolic or sigmoid curves), as appropriate. All experiments were performed in triplicate and on at least three independent occasions.

### 2.6 Adaptation of *Leishmania* promastigotes to tolerance for pyrimidine analogs

Promastigotes of the wild-type *L. mexicana* M379 and *L. major* Friedlin strains were exposed to non-lethal concentrations (0.5 × EC_50_) of 5-fluorouracil and 5-fluoro-2′-deoxyuridine. The cells were then visually observed for viability and sub-passaged to tolerated concentrations of the drugs. The procedure was repeated until a high level of tolerance to the drug was obtained, essentially as described for the adaptation of *T. brucei* to pentamidine (Bridges et al., 2007), diminazene (Teka et al., 2011), curcumin analog AS-HK014 (Changtam et al., 2010) and fluorinated pyrimidines (Ali et al., 2013a). After achieving a high level of resistance, clonal populations were obtained by limiting dilution.

### 2.7 Metabolomics sample preparation and analysis

Metabolomics analysis of the *Leishmania* promastigotes was undertaken in triplicate, exactly as described (Ali et al., 2013a). Briefly, cells were grown to log phase stage, resuspended at 2 × 10^6^ cells/ml in 50 ml HOMEM/FBS and incubated 8 h with 100 μM of test compound (standard conditions) before transfer to a 50-ml centrifuge tube for instantaneous cooling (dry ice/ethanol bath, 4 °C) and centrifugation (2500 rpm, 10 min, 4 °C). The pellet was lysed with 200 μl of chloroform/methanol/water (1:3:1 v/v/v) containing mass spectrometry standards, and vigorous mixing (1 h, 4 °C); cell debris was removed by centrifugation and the metabolite extracts were stored in HPLC vials at −80 °C. Control samples were prepared in parallel and included untreated cells, unused growth medium, test compound solution and extraction solvent blanks. The analysis used a hydrophilic interaction liquid chromatography (HILIC-LC) fitted with a zwitterionic ZIC-pHILIC column (Merck Sequant), coupled to high resolution mass spectrometry (MS) using a Thermo Q-Exactive, and metabolomic data outputs were analyzed using the IDEOM application (http://mzmatch.sourceforge.net/ideom.php) with default parameters (Creek et al., 2012) exactly as described (Ali et al., 2013a). The lower limit of detection was set to 500 intensity units for all reported metabolites, in order to prevent spurious identification of low level signals. 

## 3 Results

### 3.1 Characterization of pyrimidine transporters in promastigotes of *L. mexicana*

#### 3.1.1 [^3^H]-Thymidine uptake in *L. mexicana*

**Fig. 1:**
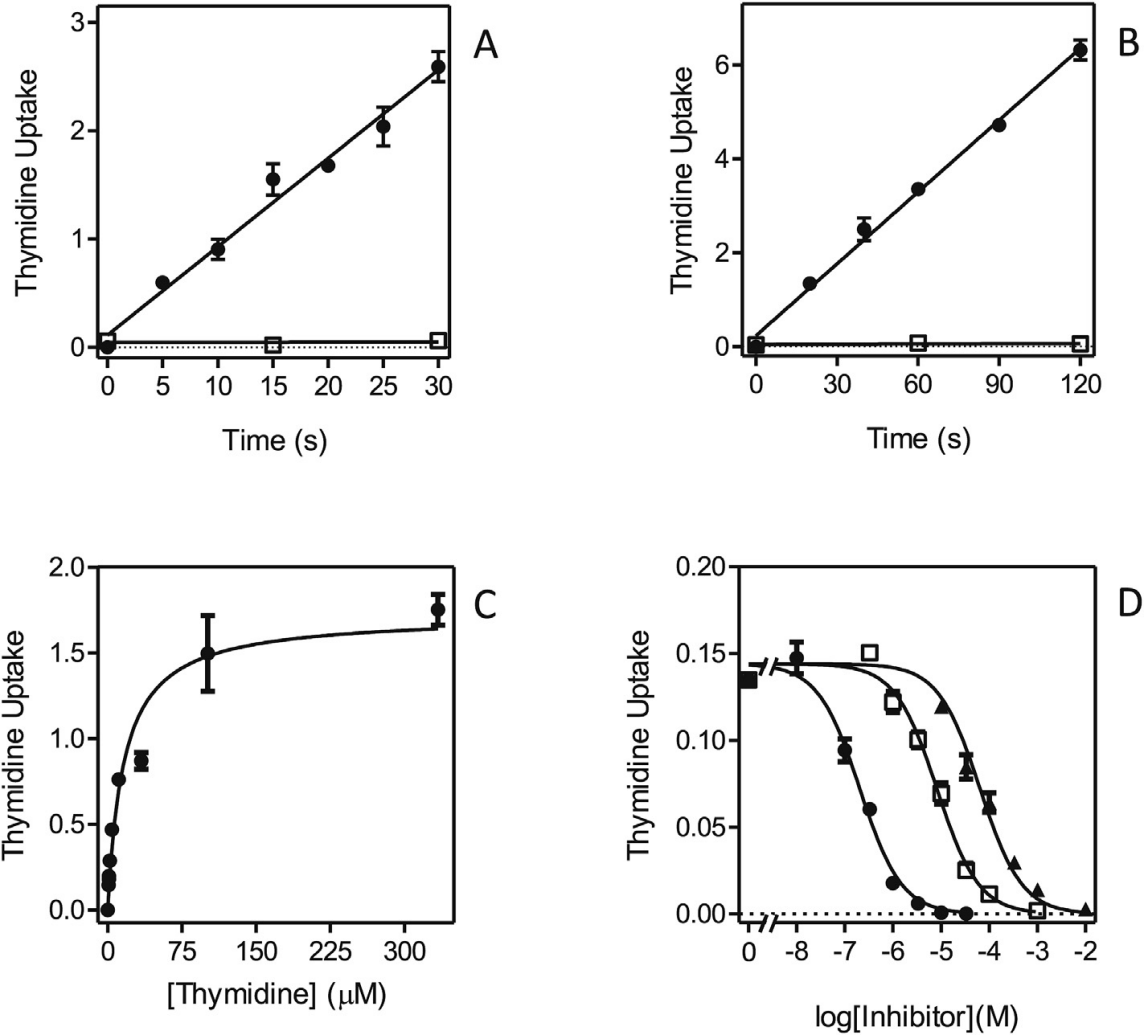
Transport of 1 μM [^3^H]-thymidine by promastigotes of *L. mexicana*. (A,B). Uptake was measured over various intervals in the presence (□) or absence of 1 mM unlabeled thymidine (●). Frame A: rate at 1 μM was 0.082 ± 0.005 pmol(10^7^ cells^)−1^s^−1^, r^2^ = 0.982, significantly non-zero *P* < 0.0001; for control with 1 mM thymidine, rate was not significantly different from zero (*P* = 0.95). Frame B: rate at 1 μM was 0.051 ± 0.002 pmol(10^7^ cells^)−1^s^−1^, r^2^ = 0.995, significantly non-zero *P* < 0.0001; for control with 1 mM thymidine, the rate was not significantly different from zero (*P* = 0.67). (C) Michaelis-Menten saturation curve for the uptake of [^3^H]-thymidine. (D) Inhibition of 1 μM [^3^H]-thymidine uptake by unlabeled adenosine (●), uridine (□) and cytidine (▲). Unit for transport was pmol(10^7^ cells)^−1^s in frames A and B, and pmol(10^7^ cells)^−1^s^−1^ in frames C and D; symbols represent the average of triplicate determinations in a single representative experiment, and error bars represent SEM.

*L. donovani* is known to express one pyrimidine nucleoside transporter, LdNT1, which also transports adenosine (Vasudevan et al., 1998, De Koning et al., 2005). In order to investigate whether this model held true for *L. mexicana*, the uptake of [^3^H]-thymidine was studied. Uptake of 1 μM [^3^H]-thymidine was linear (r^2^ = 0.98) over 30 s with a rate of 0.082 ± 0.005 pmol(10^7^ cells)^−1^s^−1^, and was clearly saturable, as transport in the presence of 1 mM unlabeled thymidine was not significantly different from zero (*P* = 0.95) (Fig. 1A). Similarly, uptake of 1 μM [^3^H]-thymidine was linear over 2 min (Fig. 1B), allowing subsequent inhibition experiments to be conducted over 20 s, very much within the linear range and thus representing the initial rate of uptake rather than a rate of metabolism. Care was taken throughout this study that whenever inhibition constants (K_i_) or Michaelis-Menten constants (K_m_) were determined the conditions used had been verified to be well within the linear range of uptake. Indeed, we did not observe non-linearity when studying nucleoside or uracil transport in *Leishmania* promastigotes, even at longer times and where there was a net-accumulation of radiolabel over the external concentration. In no time course experiments (whether shown in this paper or not) did runs tests show a significant deviation from linearity. This is likely because the rate of metabolism of nucleosides and nucleobases is very fast in kinetoplastids and therefore does not become rate limiting for uptake, especially over very short intervals. Moreover, the uptake of both nucleobases and nucleosides has been shown to be a secondary active transport mechanism, using proton symport to accumulate purines and pyrimidines highly efficiently (De Koning and Jarvis, 1997, De Koning and Jarvis, 1998, De Koning et al., 1998, Stein et al., 2003).

The Michaelis-Menten constant (K_m_) for thymidine was determined to be 11.2 ± 2.4 μM (Fig. 1C; Table 1; n = 3). This thymidine transporter was inhibited by adenosine with sub-micromolar affinity (K_i_ = 0.25 ± 0.4 μM), by uridine with low micromolar affinity (K_i_ = 9.1 ± 0.6 μM) and by cytidine with mid-micromolar affinity (K_i_ = 82 ± 5 μM) (Fig. 1D). The transporter had little or no affinity for inosine, uracil or hypoxanthine (Table 1) and is consistent with the substrate specificity of LdNT1.

**Table 1:** Transport of adenosine and pyrimidines in *Leishmania mexicana* promastigotes.

Transporter	LmexNT1			LmexUU1
radiolabel	[^3^H]-thymidine	[^3^H]-adenosine	[^3^H]-uridine^a^	[^3^H]-uracil
K_m_ (μM)	**11.2 ± 2.4**	**0.81 ± 0.16**	**13.3 ± 2.4**	**29.7 ± 4.4**
V_max_^b^	1.53 ± 0.28	1.11 ± 0.19	0.74 ± 0.13	0.088 ± 0.010
V_max_/K_m_	0.14	1.38	0.055	0.003

*inhibitors (K*_*i*_, *μM)*				
adenosine	0.25 ± 0.04	**0.81 ± 0.16**	0.39 ± 0.09	ND^c^
thymidine	**11.2 ± 2.4**	16.5 ± 0.4	9.98 ± 2.67	ND^c^
uridine	9.1 ± 0.6	16.8 ± 1.3	**13.3 ± 2.4**	2.0 ± 0.5
2′deoxyuridine	ND	ND	ND	9.3 ± 2.6
5F,2′deoxyuridine			6.99 ± 0.11	ND^c^
cytidine	82 ± 5	149 ± 18	78.6 ± 15.1	ND
inosine	640 ± 34	1630 ± 134	1010 ± 190	ND
uracil	>2500	>2500	>2500	**29.7 ± 4.4**
5FU	ND	ND	>5000	56.3 ± 6.4
thymine	ND	ND	ND	560 ± 190
cytosine	ND	ND	ND	>5000
hypoxanthine	>1000	ND	>1000	>500
adenine	ND	>1000	>1000	288 ± 67

For each radiolabel, K_m_ values are listed in bold typeface and reproduced in the inhibitors table for easy reference.

ND, not done.

a Measured in the presence of 1 mM uracil.

b Expressed in pmol(10^7^ cells)^−1^s^−1^.

c These values are estimated to be similar to those of LmexNT1, based on the observation that the Hill slope for inhibition of [^3^H]-uridine was approximately −1, and the level of inhibition 100%.

#### 3.1.2 [^3^H]-Adenosine uptake in *L. mexicana*

**Fig. 2:**
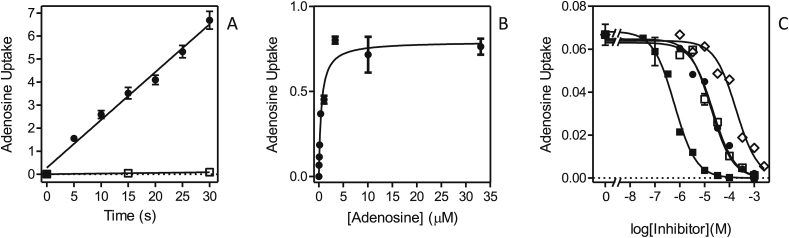
Transport of [^3^H]-adenosine by *L. mexicana* promastigotes. (A) Transport of 0.1 μM [^3^H]-adenosine in the presence (□) or absence of 1 mM unlabeled adenosine (●). Rate at 0.1 μM was 0.21 ± 0.01 pmol(10^7^ cells^)−1^s^−1^, r^2^ = 0.988, significantly non-zero *P* < 0.0001; for control with 1 mM adenosine, the rate was 0.0028 ± 4.410^−6^ pmol(10^7^ cells^)−1^s^−1^ (*P* = 0.001). (B) Michaelis-Menten saturation curve for the uptake of [^3^H]-adenosine using 0.05 μM radiolabel and various concentrations of unlabeled adenosine up to 33.3 μM. (C) Inhibition of 0.05 μM [^3^H]-adenosine uptake by unlabeled adenosine (■), thymidine (□), uridine (●) and cytidine (◊). Unit for transport was pmol(10^7^ cells)^−1^ for frame A, and pmol(10^7^ cells)^−1^s^−1^ in frames B and C; symbols represent the average of triplicate determinations in a single representative experiment, and error bars represent SEM.

We next investigated the transport of [^3^H]-adenosine in *L. mexicana* promastigotes, which according to the efficient inhibition of [^3^H]-thymidine by adenosine, should be taken up with high affinity. Transport of 0.1 μM [^3^H]-adenosine was linear over at least 30 s with a rate of 0.21 ± 0.01 pmol(10^7^ cells)^−1^s^−1^ (Fig. 2A) and the K_m_ value was determined as 0.81 ± 0.16 μM (Fig. 2B). Fig. 2C shows the inhibition curves for adenosine (high affinity), uridine and thymidine (almost identical, low micromolar), and cytidine (mid-micromolar affinity). Adenosine transport was not inhibited by adenine or uracil and only with very low affinity by inosine (1.6 ± 0.1 mM; Table 1). The kinetics showed no indication for more than 1 transport activity for adenosine (100% inhibition by pyrimidine nucleosides; Hill slopes consistently near −1).

#### 3.1.3 [^3^H]-uridine uptake in *L. mexicana*

**Fig. 3:**
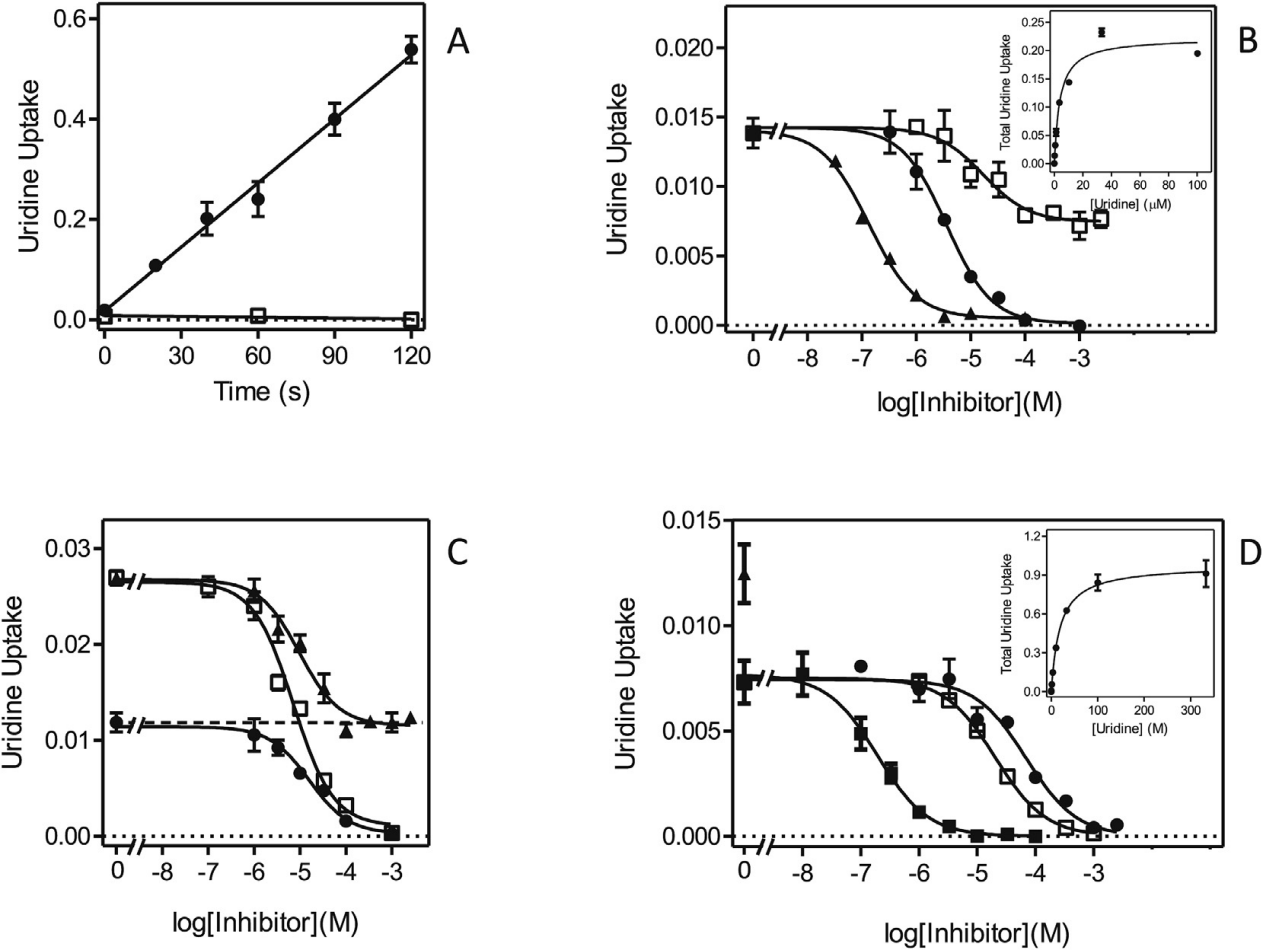
Transport of [^3^H]-uridine by *L. mexicana* promastigotes. (A) Transport of 0.25 μM [^3^H]-uridine was linear (r^2^ = 0.992) over 120 s with a rate of 0.0043 ± 0.0002 pmol(10^7^ cells^)−1^s^−1^ (●). In the presence of 5 mM uridine (□) transport was not significantly different from zero (*P* = 0.43). (B) Transport of 0.25 μM [^3^H]-uridine was dose-dependently inhibited by unlabeled uridine (●), resulting in an apparently mono-phasic sigmoid curve that could be converted to a Michaelis-Menten saturation plot (*inset*) to determine K_m_ and V_max_ values. Transport was inhibited by 48% by up to 2.5 mM uracil (□), and ∼100% by adenosine (▲). (C) Transport of 0.25 μM [^3^H]-uridine was determined in the presence (●) or absence (□,▲) of 1 mM unlabeled uracil in order to inhibit the uracil-sensitive component of uridine uptake. Inhibitors shown are uracil (▲) and thymidine (□,●). The dotted line indicates the level of uridine uptake in the presence of 1 mM uracil with zero thymidine added. (D) Transport of 0.25 μM [^3^H]-uridine in the presence of 1 mM uracil was dose-dependently inhibited by adenosine (■), uridine (□) and cytidine (●). The uridine inhibition data was converted to a Michaelis-Menten saturation plot (*inset*). The level of [^3^H]-uridine in the absence of uracil or other inhibitor is also indicated (▲). Unit for transport was pmol(10^7^ cells)^−1^ for frame A and pmol(10^7^ cells)^−1^s^−1^ in frames B-D; symbols represent the average of triplicate determinations in a single representative experiment, and error bars represent SEM.

Fig. 3A shows that 0.25 μM [^3^H]-uridine was taken up efficiently and linearly over 120 s, with a rate of 0.0042 ± 0.0002 pmol(10^7^ cells)^−1^s^−1^. The K_m_ for the uridine flux was determined to be 7.15 ± 0.90 μM (n = 5). As shown in Fig. 3B, the Hill slope for inhibition of [^3^H]-uridine uptake by uridine was close to −1 (−0.91 ± 0.09, n = 5), and thus consistent with a one-transporter model, but uracil inhibited only part of the flux, revealing the existence of a uracil-sensitive transporter (K_i_ = 25.7 ± 6.6 μM (n = 5)) and a uracil-insensitive uridine transporter. From the [^3^H]-uridine Hill slope it follows that both transporters have a similar affinity for uridine and Fig. 3C furthermore shows that both are similarly sensitive to thymidine, which was able to inhibit 100% of uridine transport. The uracil-insensitive transport could be studied in isolation by the inclusion of 1 mM uracil in the transport assay buffer, blocking the uracil-sensitive component (Fig. 3C), revealing that indeed the EC_50_ for thymidine was similar for the uracil-sensitive and –insensitive uridine transporters. Adenosine clearly inhibited both transporters with near-equal affinity, with a Hill slope close to −1 (Fig. 3B). The K_m_ of the uracil-insensitive transporter was subsequently determined to be 13.3 ± 2.4 μM, and its inhibition profile, showing high affinity for adenosine and mid-micromolar affinity for cytidine (Fig. 3D) clearly established that this component is mediated by the same transporter as that mediating thymidine and adenosine uptake. This is thus, like LdNT1, an adenosine/uridine/thymidine transporter, insensitive to purine or pyrimidine nucleobases or to sub-millimolar concentrations of inosine (Table 1), and we hereby designate it LmexNT1.

#### 3.1.4 [^3^H]-Uracil uptake in *L. mexicana*

**Fig. 4:**
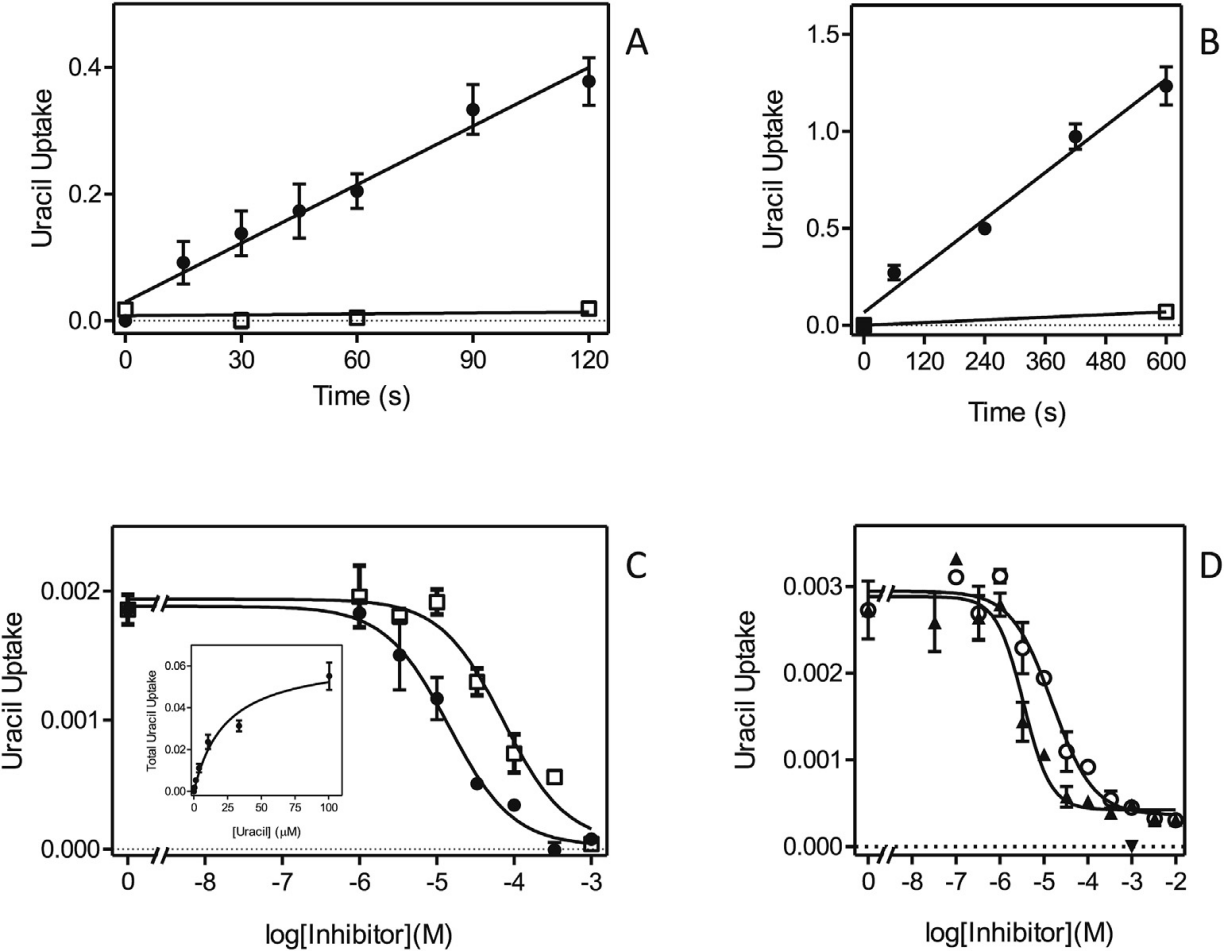
Transport of [^3^H]-uracil by *L. mexicana* promastigotes. (A) Transport of 1 μM [^3^H]-uracil was linear over 120 s with a rate of 0.0030 ± 0.0002 pmol(10^7^ cells)^−1^s^−1^ (●, r^2^ = 0.974; *P* < 0.0001) and was fully inhibited by 1 mM unlabeled uracil (□, not significantly different from zero, *P* = 0.73). (B) Transport of 0.5 μM [^3^H]-uracil was linear over 10 min with a rate of 0.0020 ± 0.0002 pmol(10^7^ cells)^−1^s^−1^ (●, r^2^ = 0.981; *P* = 0.0011) and was 95% inhibited by 1 mM unlabeled uracil. (C) Transport of 0.5 μM [^3^H]-uracil was dose-dependently inhibited by uracil (●) and by 5-fluorouracil (□). The inhibition data for unlabeled uracil were converted to a Michaelis-Menten saturation plot (*inset*). (D) Inhibition of 0.5 μM [^3^H]-uracil transport by uridine (▲) and by 2′-deoxyuridine (○). The level of inhibition by 1 mM unlabeled uracil is indicated (▼). Unit for transport was pmol(10^7^ cells)^−1^ for frames A and B, and pmol(10^7^ cells)^−1^s^−1^ in frames C and D; symbols represent the average of triplicate determinations in a single representative experiment, and error bars represent SEM.

In order to study the uracil-sensitive uridine transporter we next employed 1 μM [^3^H]-uracil, which was taken up linearly over 120 s (Fig. 4A) and we first attempted to characterize the flux over just 20 s, but found that the relatively low levels of uptake resulted in poor resolution of inhibition curves. We therefore extended the time course and found that at the reduced concentration of 0.25 μM linearity extended to at least 10 min (Fig. 4B), with a rate of 0.0020 ± 0.0002 pmol(10^7^ cells)^−1^s^−1^. Transport of uracil over 4 min could thus be measured accurately and the K_m_ was determined as 29.7 ± 4.4 μM (n = 3), with 5-fluorouracil displaying a somewhat lower affinity with a K_i_ of 56.3 ± 4.4 μM (n = 3, *P* < 0.05) (Table 1 and Fig. 4C). Consistent with the description of a uracil-sensitive uridine transporter, above, [^3^H]-uracil transport was dose-dependently inhibited by uridine (K_i_ = 2.0 ± 0.5 μM), as well as by 2′-deoxyuridine (K_i_ = 9.3 ± 2.6 μM; *P* < 0.05) (Fig. 4D). However, it was consistently observed that both of these substrates, unlike uracil and 5-fluorouracil, inhibited only approximately 85% of [^3^H]-uracil transport (n = 3). However, the flux through a presumed uridine-insensitive uracil transporter was too small to be characterized; it could be speculated that it is barely expressed in the promastigote forms, at least under the culture conditions used. We propose the designation *L. mexicana* uridine-uracil transporter 1 (LmexUUT1) for the uracil-sensitive uridine transporter here described.

### 3.2 Characterization of pyrimidine transporters in promastigotes of *L. major*

#### 3.2.1 Thymidine transport in *L. major*

**Fig. 5:**
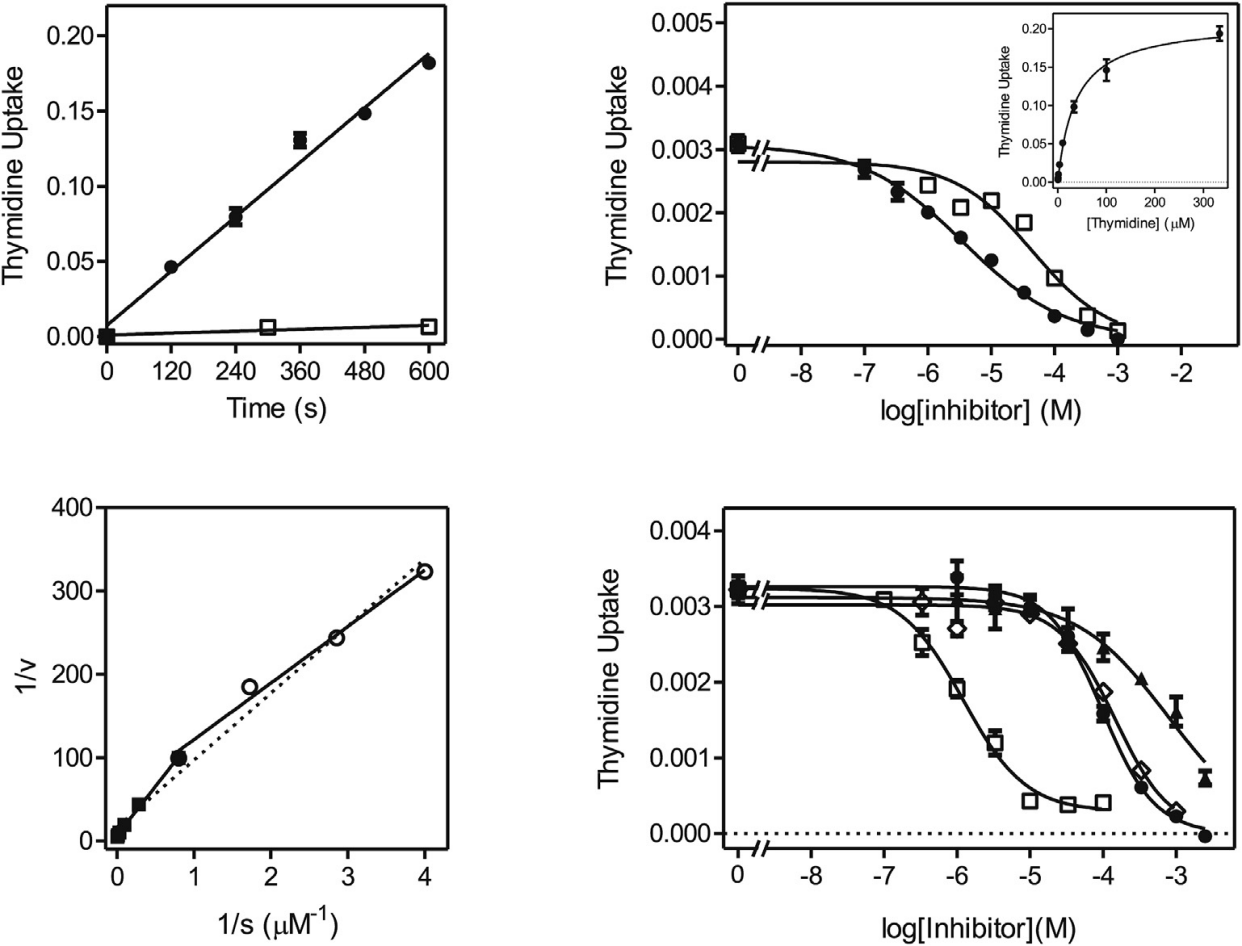
Transport of 0.25 μM [^3^H]-thymidine by *L. major* promastigotes. (A) Transport of 0.25 μM [^3^H]-thymidine was linear for 10 min with a rate of 0.00030 ± 0.00002 pmol(10^7^ cells)^−1^s^−1^ (●, r^2^ = 0.986; *P* < 0.0001). In the presence of 1 mM unlabeled thymidine, transport was completely inhibited (□, not significantly different from zero, *P* = 0.30). (B) Transport of [^3^H]-thymidine was inhibited by unlabeled uridine (□) and thymidine (●). The latter inhibition curve was also converted to a Michaelis-Menten saturation curve (*inset*). (C) Lineweaver-Burk double reciprocal plot of the thymidine inhibition plot in panel B, showing the separation into two distinct transport activities, a higher affinity transport component with apparent K_m_ 1.26 μM (○) and a lower affinity component with apparent K_m_ 17.1 μM in this experiment (■). Inhibition plots for the transport of 0.25 μM [^3^H]-thymidine by adenosine (□), 4-thiouridine (●), 2′deoxyuridine (◊) and cytidine (▲). Unit for transport was pmol(10^7^ cells)^−1^ for frame A and pmol(10^7^ cells)^−1^s^−1^ in frames B-D; symbols represent the average of triplicate determinations in a single representative experiment, and error bars represent SEM.

Transport of 0.25 μM [^3^H]-thymidine was linear over 10 min, albeit with a very low rate of 0.0043 ± 0.0002 pmol(10^7^ cells^)−1^s^−1^, and completely inhibited by 1 mM unlabeled thymidine (Fig. 5A). Subsequent experiments were performed with a 5 min incubation time. Fig. 5B shows representative inhibition curves for the inhibition of [^3^H]-thymidine transport by unlabeled uridine and thymidine. Thymidine was the stronger inhibitor and, calculated from the Michaelis-Menten saturation plots (unlabeled thymidine in competition with [^3^H]-thymidine) (Fig. 5B, *inset*), an average K_m_ value of 30.7 ± 2.1 μM (n = 5) was calculated. This compared to a K_i_ value of 61.1 ± 7.4 μM (n = 3) for uridine. The figure further illustrates that the Hill coefficient of both sigmoid curves was greater than the value of −1 associated with a one-transporter model. The average Hill slopes were −0.67 ± 0.06 and −0.87 ± 0.10 for thymidine and uridine, respectively – indicative of a two-component transport system with distinct but not greatly different affinities. In order to separate out the two transporters, the thymidine inhibition data were redrawn using a Lineweaver-Burke double reciprocal plot (Fig. 5C), revealing a high affinity thymidine transporter with an average K_m_ of 4.2 ± 1.1 μM and V_max_ of 0.023 ± 0.003 pmol(10^7^ cells^)−1^s^−1^, and a lower affinity thymidine transporter with a K_m_ of 26.8 ± 5.4 μM and V_max_ of 0.14 ± 0.03 pmol(10^7^ cells^)−1^s^−1^ (both n = 5). The approximately 6-fold difference in apparent K_m_ between the two transporters would be consistent with the observed Hill slopes. Indeed, we were unable to separate the two transporters by inhibitor profile, with several inhibitors displaying 100% inhibition and Hill coefficients near −1, or incomplete inhibition at the highest concentration tested (Fig. 5D). Adenosine was the highest affinity inhibitor (K_i_ = 1.77 ± 0.21 μM, n = 4) and in most cases inhibited 100% of [^3^H]-thymidine transport, although in some cases a small percentage of the flux appeared to resist adenosine inhibition (Fig. 5D). Our interpretation of these results is that the two slightly different thymidine transport activities here observed represent LmajNT1.1 and LmajNT1.2 (see below).

Table 2 presents an overview of inhibitors of [^3^H]-thymidine transport in *L. major* promastigotes, which we treat as inhibitors of the total thymidine transport activity constituted of LmajNT1.1 and LmajNT1.2, as we were unable to measure each separately in wild-type promastigotes and the kinetics strongly suggested that both transporters were similarly sensitive to each inhibitor. Apart from adenosine, Fig. 5D shows inhibition by several uridine analogs: 4-thiouridine and 2′-deoxyuridine displayed highly similar Ki values to uridine (Table 2), showing that neither the 2′ hydroxyl nor the 4-position keto group of uridine are directly involved in interactions with the transporter binding pocket. However, 2-thiouridine displayed much lower affinity for the NT1 transporters (Fig. 5D), with a K_i_ value of 765 ± 68 μM versus 55.7 ± 5.4 μM for uridine. When these values are converted to the Gibbs free energy of binding (ΔG^0^), it can be found that the difference in binding energy (δ(ΔG^0^)) is 6.5 kJ/mol (Table 2), which can be attributed to an interaction with the 2-position keto group with the transporter binding site, following a method previously used to construct binding models for other protozoan and human transporters (De Koning and Jarvis, 1999, Wallace et al., 2002, De Koning et al., 2003, Al-Salabi et al., 2007). Following similar reasoning, it can be concluded that the 3-position pyrimidine nitrogen is also involved in a positive interaction with the binding site, as 3-deazauridine displayed much-reduced binding energy (δ(ΔG^0^) = 10.4 kJ/mol). Furthermore, cytidine also displayed low affinity, but, since the 4-position keto group is not involved in binding, this can be attributed to the change in N(3) protonation state in cytidine versus uridine, consistent with a role for N(3). Substitutions at position 5 of the pyrimidine ring (methyl, Fluor) appear to be slightly favorable (Table 2) but more importantly the 3′ and 5′ hydroxyl groups of the ribose moiety are clearly involved in binding, given a δ(ΔG^0^) of 14.1 kJ/mol comparing 3′-deoxythymidine with thymidine, and 4.9 kJ/mol comparing 5-fluorouridine with 5-fluoro, 5′-deoxyuridine, respectively. The contribution of 2 strong interactions from the ribose moiety to pyrimidine nucleoside binding to the NT1 transporters provides the rationale for the complete lack of inhibition by up to 1 mM of the nucleobases adenine, uracil and thymine (Table 2). Thus, four interactions between the *L. major* NT1 transporter and uridine can be identified, and these account energetically for the uridine ΔG^0^: with keto position 2, nitrogen on position 3, and the hydroxyl groups at positions 3′ and 5′. Three of those interactions (N3, 3′OH and 5′OH) were also observed in the binding mode for uridine for the *Toxoplasma gondii* AT2 and *Trypanosoma brucei* P1 transporters (De Koning et al., 2003). In the case of TgAT2, the transporter also engaged in a π-stacking interaction with the substrate, and in the case of TbP1, no other interactions could be demonstrated, explaining the relatively low affinity for pyrimidines. TgAT2 also interacted with the nitrogen on position 3 of the purine ring, explaining its affinity for both pyrimidine and purine nucleosides, and indeed for both oxopurines inosine and guanosine and the aminopurine adenosine (De Koning et al., 2003). From this example it follows that LmajNT1 is likely to form a productive hydrogen bond with the 6-position amine of adenosine and/or with the protonated N1 residue - either would explain its selectivity for aminopurines, as previously demonstrated for the *T. brucei* P2 transporter (Munday et al., 2015).

**Table 2:** Transport of pyrimidine nucleosides in *Leishmania major*.

radiolabel	LmajUUT1	LmajNT1			
	[^3^H]-uridine	[^3^H]-uridine	[^3^H]-thymidine		
	K_m_ or K_i_ (μM)	K_m_ or K_i_ (μM)	K_m_ or K_i_ (μM)	ΔG^0^	δ(ΔG^0^)
K_m_ (1)	3.12 ± 0.61		4.20 ± 1.09	−30.7	
V_max_ (1)^a^	0.036 ± 0.004		0.023 ± 0.003		
K_m_ (2)		33.5 ± 7.3	26.9 ± 5.4	−26.9	
V_max_ (2)^a^		0.15 ± 0.04	0.14 ± 0.03		

*Inhibitors (K*_*i*_, *μM)*					
Uridine			55.7 ± 5.4	−24.3	−6.4 [tmd]
Thymidine	60.9 ± 8.4				
Cytidine			1150 ± 108	−16.8	−7.5 [urd]
Adenosine	1.93 ± 0.49		1.77 ± 0.21	−32.8	2.1 [tmd]
Inosine	0.50 ± 0.16	>1000	1990 ± 3050	−15.4	−17.4 [ado]
5-fluorouridine			23.4 ± 1.3	−26.4	2.1 [urd]
5-fluoro,2′-deoxyuridine			29.7 ± 2.1	−25.8	−0.6 [5F-urd]
5-fluoro,5′-deoxyuridine			168 ± 32	−21.5	−4.9 [5F-urd]
2′-deoxyuridine	24.9 ± 8.6		112 ± 17	−22.5	−1.7 [urd]
3′-deoxythymidine			1246 ± 44.5	−16.6	−14.1 [tmd]
2′,3′-dideoxyuridine			>2500	>-15	<-9.3 [urd]
2-thiouridine			765 ± 68	−17.8	−6.5 [urd]
4-thiouridine			69.0 ± 18.1	−23.8	−0.5 [urd]
3-deazauridine			3670 ± 420	−13.9	−10.4 [urd]
thymine			>1000		
Uracil	2.65 ± 0.60	>1000	>1000		
adenine	5.14 ± 2.25	>1000	>1000		

a Expressed in pmol(10^7^ cells)^−1^s^−1^.

#### 3.2.2 [^3^H]-Uridine transport in *L. major*

**Fig. 6:**
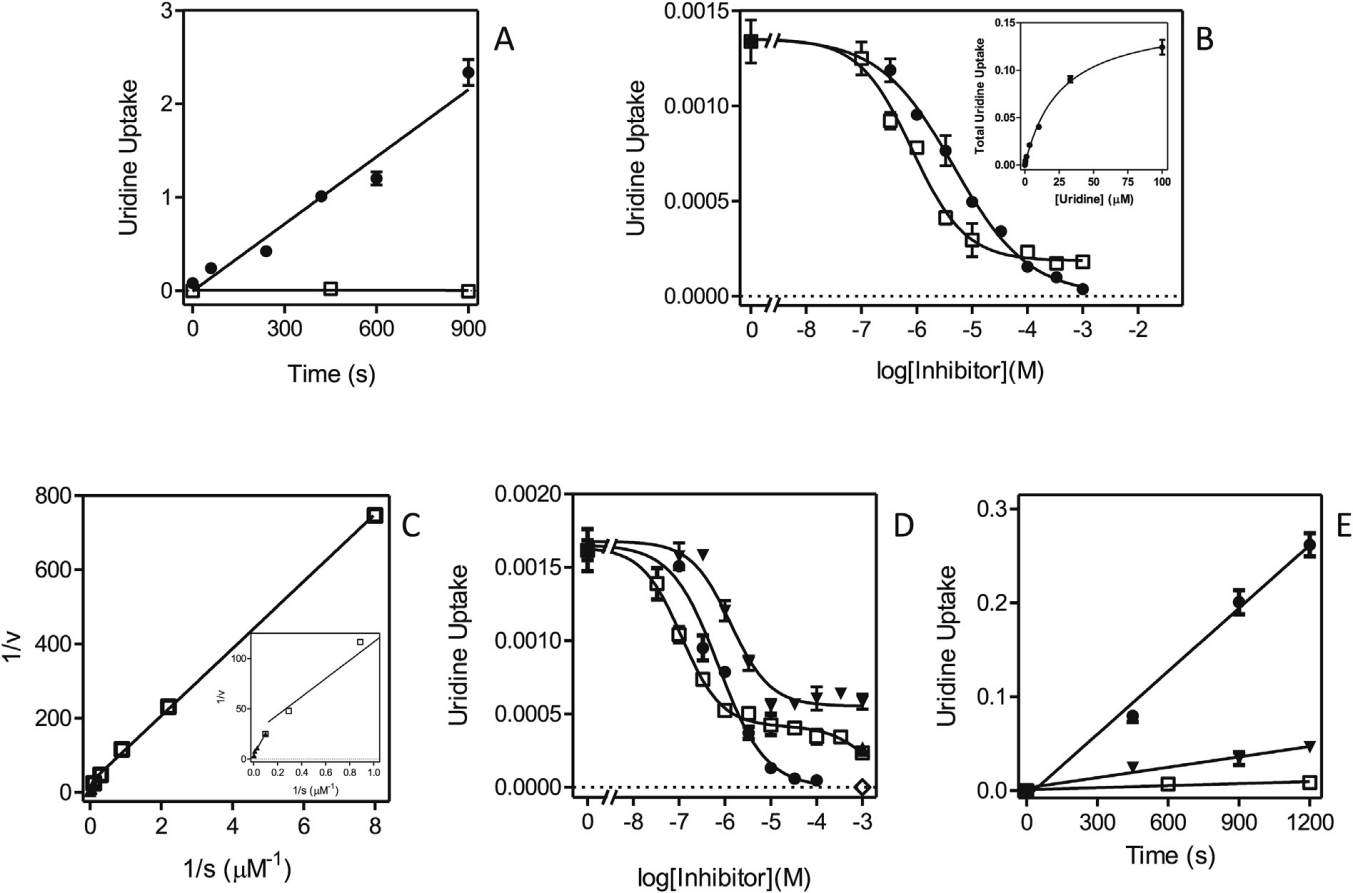
Transport of 0.25 μM [^3^H]-uridine by *L. major* promastigotes. (A) Uptake of [^3^H]-uridine in the presence (□) or absence (●) of 1 mM unlabeled uridine. The rate of uptake at 0.25 μM uridine was determined as 0.0024 ± 0.0002 pmol(10^7^ cells)^−1^s^−1^ by linear regression (r^2^ = 0.964; *P* = 0.0005), but was not significantly different from zero (*P* = 0.94) in the presence of 1 mM unlabeled permeant. (B) Sigmoid inhibition curves for uridine (●) and uracil (□). The former was converted to a Michaelis-Menten saturation curve (*inset*). (C) Conversion of the uridine inhibition data of panel B to a Lineweaver-Burk double reciprocal plot, showing separate linear regression lines for the high affinity (□, r^2^ = 0.999) and low affinity (▲, r^2^ = 0.981) components. *Inset*: zoom-in of main plot. (D) Sigmoid inhibition plots with inosine (□), adenosine (●) and adenine (▼). Also shown are individual points showing the level of inhibition with 1 mM uridine (◊) and with 1 mM adenine + 1 mM inosine (▲). (E) Time course of 0.25 μM [^3^H]-uridine transport over 20 min in the presence of 100 μM inosine (▼, r^2^ = 0.967, rate significantly different from zero *P* = 0.017 (F-test)), in the presence of 1 mM uridine (□, rate not significantly different from zero, *P* = 0.22 (F-test)) or without any inhibitors (●, r^2^ = 0.993, rate significantly different from zero *P* = 0.0035 (F-test)). Unit for transport was pmol(10^7^ cells)^−1^ for frames A and E, and pmol(10^7^ cells)^−1^s^−1^ in frames B-D; symbols represent the average of triplicate determinations in a single representative experiment, and error bars represent SEM.

Transport of 0.25 μM [^3^H]-uridine in *L. major* promastigotes proceeded at approximately half the rate of [^3^H]-thymidine transport at the same concentration: Fig. 6A shows linear uptake of 0.25 μM [^3^H]-uridine over 15 min at a rate of 0.0024 ± 0.0002 pmol(10^7^ cells^)−1^s^−1^. Like [^3^H]-thymidine transport, two distinct components for [^3^H]-uridine transport were in evidence, leading to a Hill coefficient of −0.79 ± 0.11 for inhibition with unlabeled uridine (Fig. 6B). As for thymidine transport, it was possible to convert the inhibition plot to a single Michaelis-Menten saturation plot (which gave an apparent K_m_ of 7.3 ± 1.3 μM (n = 3)) (Fig. 6B, *inset*), but a double reciprocal plot separated two components (Fig. 6C) with apparent K_m_ values of 3.1 ± 0.6 μM and 33.5 ± 7.3 μM (both n = 3) (Table 2). Moreover, several inhibitors displayed only partial inhibition, of just the high-affinity transporter, whereas several other inhibitors appeared to inhibit both with similar affinities. Uracil, for instance, inhibited only 77.2 ± 2.7%, with an average Hill coefficient of −0.93 ± 0.01(n = 4) – indicative of a single transport system (Fig. 6B). Similar observations (Fig. 6D) were made for adenine (62.7 ± 1.7% inhibition; Hill coefficient is −1.25 ± 0.16) and inosine (Hill coefficient −1.09 ± 0.04; 70.8 ± 4.5% inhibition at 100 μM). Inosine did appear to start to slightly inhibit the second transport component at millimolar concentrations, but crucially the inhibition of 1 mM each of adenine and inosine was not additive, proving that both fully inhibited the same transporter (Fig. 6D). The partial inhibition of [^3^H]-uridine by inosine was further confirmed by a time course over 20 min (Fig. 6E), in which 100 μM inosine inhibited 0.25 μM uridine transport by 83.5% (n = 2) but the slope of time-dependent uridine uptake in the presence of inosine was significantly non-zero (*P* = 0.017), compared to the complete inhibitory effect of 1 mM unlabeled uridine (*P* = 0.22).

It is thus clear that *L. major* promastigotes, like *L. mexicana*, express a uracil-sensitive uridine transporter, and accordingly we designate this activity LmajUUT1. Both *L. major* uridine transport activities were sensitive to the nucleosides thymidine and adenosine and 2′-deoxyuridine (Table 2). Fig. 6D shows the complete inhibition of [^3^H]-uridine transport by adenosine, with a K_i_ value of 1.93 ± 0.49 μM. We conclude that *L. major* expresses two similar adenosine/thymidine/uridine transporters, one of which is sensitive to inhibition by uracil, adenine and inosine.

### 3.3 Molecular cloning and functional characterization of the *L. major* and *L. mexicana* NT1 and NT2 nucleoside transporters

Open reading frames encoding members of the Equilibrative Nucleoside Transporter family were identified in the *L. mexicana* and *L. major* genomes (http://www.genedb.org/). The syntenic genes to *L. donovani* NT1.1, NT1.2 and NT2 were cloned and introduced into the clonal *T. brucei* cell line B48, which lacks both the aminopurine transporter TbAT1 and the High Affinity Pentamidine Transporter HAPT1 (Bridges et al., 2007) using the expression vector pHD1336 as described (Munday et al., 2013, Munday et al., 2015). Correct integration of the linearized construct into the *T. brucei* genome was confirmed by PCR, and expression of all six genes (Table S1) was confirmed using qRT-PCR (Supplemental Fig. 1). Analysis of NT1 and NT2 expression in promastigotes versus amastigotes of *L. mexicana* revealed that both transporter types were similarly expressed in these life cycle stages (Supplemental Fig. 2).

#### 3.3.1 Functional characterization of the NT1A and NT1B transporters

**Fig. 7:**
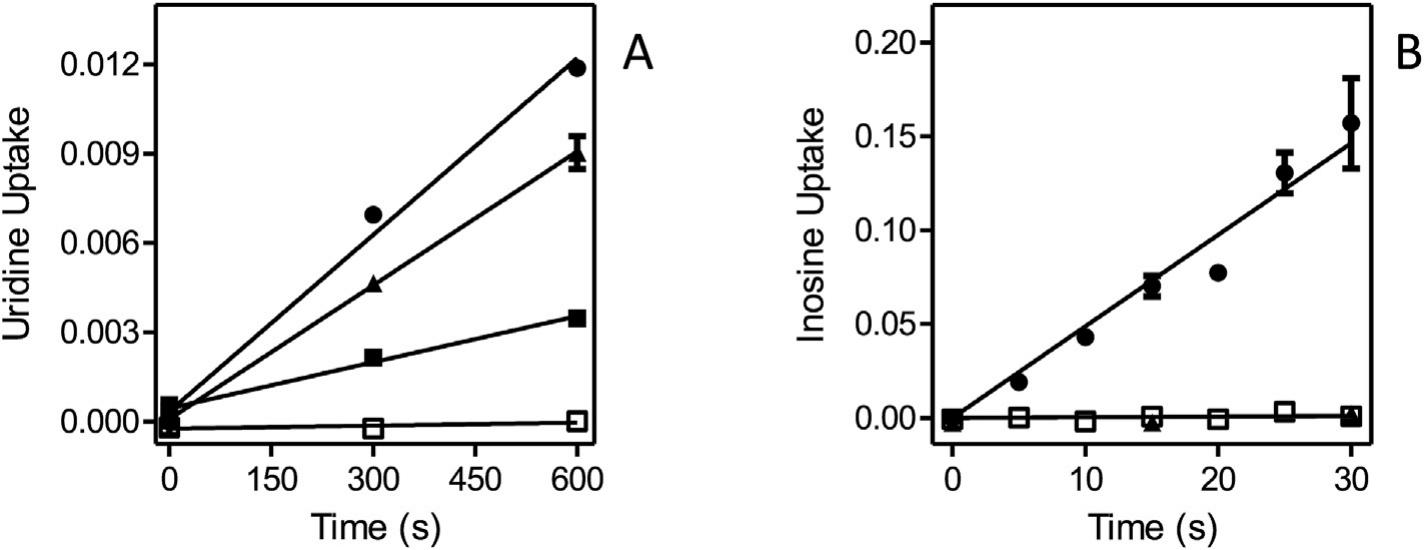
Uridine and inosine transport in *T. b. brucei* strain B48. (A) Uptake of 0.5 μM [^3^H]-uridine (●) was inhibited 24.3% by 250 μM inosine (▲) and 73.9% by uracil (■); the two inhibitors combined 100% inhibited [^3^H]-uridine uptake (□), as did 1 mM unlabeled uridine (not shown for reasons of clarity). (B) Transport of 50 nM [^3^H]-inosine (●) was fully inhibited by either 250 μM adenosine (□) or 1 mM unlabeled inosine (▲). Unit for transport was pmol(10^7^ cells)^−1^ in both frames; symbols represent the average of triplicate determinations in a single representative experiment, and error bars represent SEM.

The activity of the *Leishmania* NT1 and NT2 transporters in *T. brucei* clone B48 was assessed using [^3^H]-uridine and [^3^H]-inosine, respectively. We first assessed uridine and inosine uptake in the non-transfected cells. Fig. 7A shows that uptake of 0.5 μM [^3^H]-uridine in *T. brucei* B48 was very slow, with a rate of just 1.98 × 10^−5^ ± 1.9 × 10^−6^ pmol(10^7^ cells)s^−1^, and was strongly inhibited (75.7%) by 250 μM uracil, and to a lesser extent by inosine (24.3%). The combination of inosine and uracil fully inhibited the [^3^H]-uridine transport. This is consistent with our previous characterizations of purine and pyrimidine transporters in *T. brucei*, showing that uridine can be taken up, although very inefficiently, by the U3 uracil transporter (Ali et al., 2013a, Ali et al., 2013b), and also has a low affinity (K_i_ = 830 μM) for the P1 adenosine/inosine transporter (De Koning and Jarvis, 1999). [^3^H]-inosine was transported far more robustly by these cells, with the low concentration of 50 nM taken up at 0.0049 ± 0.0002 pmol(10^7^ cells)s^−1^ (Fig. 7B); the transport was completely inhibited by 1 mM of either unlabeled inosine or adenosine, consistent with our previous reports that inosine is only taken up by the P1 purine nucleoside transporters (De Koning et al., 1998, De Koning and Jarvis, 1999, Al-Salabi et al., 2007, Munday et al., 2013).

**Fig. 8:**
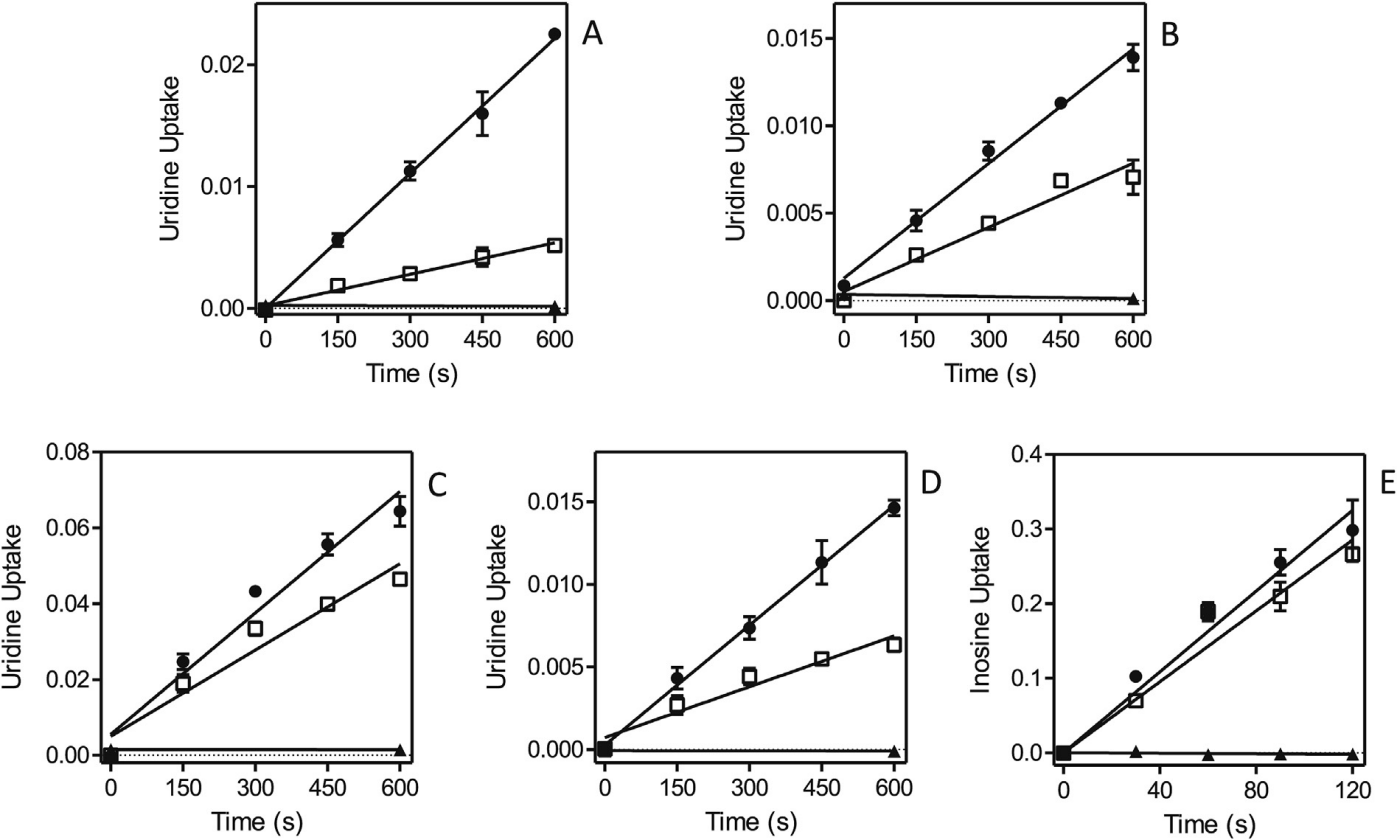
Uridine and inosine transport in *T. brucei* B48 transfected with *Leishmania* NT1-type transporters. (A) B48 transfected with LmexNT1A. Transport of 0.5 μM [^3^H]-uridine (●) was partly inhibited by a combination of 250 μM uracil and 250 μM inosine (□); inhibition was complete with 1 mM uridine (▲). (B–D) As (A) but for LmajNT1A, LmexNT1B and LmajNT1B, respectively. (E) Transport of 50 nM [^3^H]-inosine by B48 transfected with LmajNT1A (●) was slightly inhibited by 250 μM uridine (□) and completely by 250 μM adenosine (▲) as well as by 1 mM inosine (not shown for clarity). Unit for transport was pmol(10^7^ cells)^−1^ in all frames; symbols represent the average of triplicate determinations in a single representative experiment, and error bars represent SEM.

Each of the *Leishmania* NT1-type transporters, LmexNT1A/B and LmajNT1A/B, were separately transfected into B48 and [^3^H]-uridine transport (Fig. 8A–D). In each case, uridine transport was mediated by three transport activities: the *T. brucei* transporters U3 and P1, and the heterologous *Leishmania* transporter. As the *Leishmania* NT1 transporters are not inhibited by either uracil or inosine, the combination of both was used to block all endogenous [^3^H]-uridine transport, and the remaining uridine uptake represented uridine uptake by the *Leishmania* transporter; the rate of uridine uptake by U3+P1 follows from the subtraction of the uninhibited rate with the rate in the presence of uracil and inosine. All four heterologous transporters mediated uridine transport (Fig. 8A–D), and in each case the transport was fully inhibited by 250 μM adenosine (Supplemental Fig. S3). In the experiments shown, LmexNT1B displayed the highest rate of [^3^H]-uridine transport. However, since this is a complex function of its expression levels, translation efficiency and correct cellular localization among other factors, it would not be right to assert, based solely on these data, that this transporter is a more efficient uridine transporter than the others. It was verified for one sample transporter, LmajNT1, that it did not mediate the transport of [^3^H]-inosine (Fig. 8E). In this experiment, transport of 50 nM [^3^H]-inosine was assessed in the presence and absence of 250 μM uridine, which fully inhibits the *Leishmania* NT1 transporters, but has little or no effect on either the P1 or U3 transporters (see above). As uridine had only a marginal effect on [^3^H]-inosine transport in the cells expressing LmajNT1A, this transporter did not mediate any substantial amounts of inosine uptake.

#### 3.3.2 Functional characterization of the *Leishmania* NT2 transporters expressed in B48

**Fig. 9:**
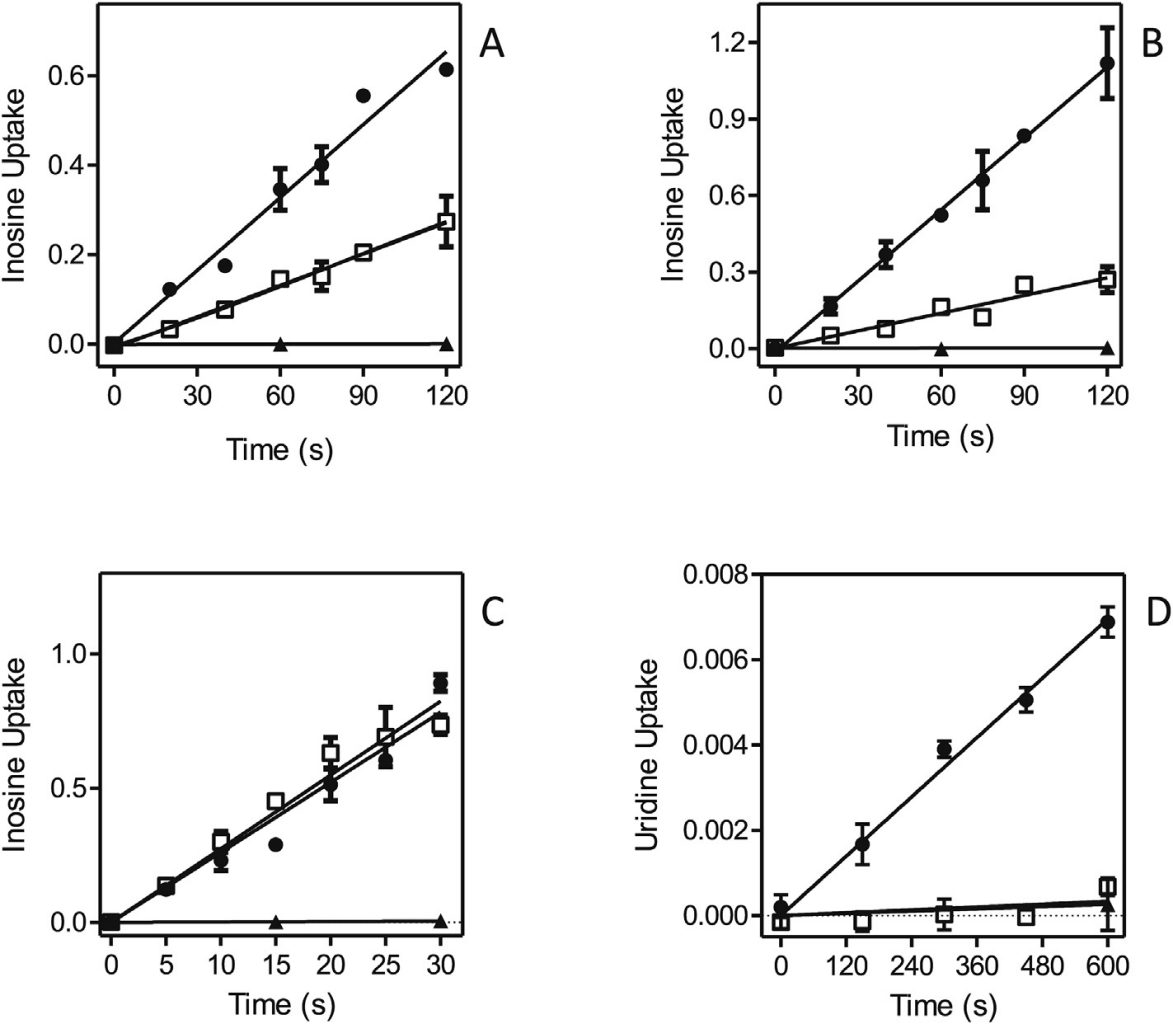
Nucleoside transport by *T. brucei* B48 cells transfected with *Leishmania* NT2 transporters. (A) Transport of 50 nM [^3^H]-inosine by B48 cells expressing LmajNT2 (●). Transport was 57% inhibited by 250 μM adenosine (□). Uptake in the presence of adenosine was 0.0023 ± 0.0001 pmol(10^7^ cells)^−1^s^−1^ (slope significantly different from zero *P* < 0.0001, F-test; r^2^ = 0.99), and 99.8% inhibited in the presence of 1 mM inosine (▲; not significantly different from zero, *P* > 0.05). (B) As frame (A), with LmexNT2. The rate of transport in the presence of adenosine was 0.0023 ± 0.0003 pmol(10^7^ cells)^−1^s^−1^ (slope significantly different from zero *P* = 0.0008, F-test; r^2^ = 0.91), and 99.9% inhibited in the presence of 1 mM inosine (▲; not significantly different from zero, *P* > 0.05). (C) Transport of 50 nM [^3^H]-inosine by *L. mexicana* promastigotes (●), in the presence of added 1 mM adenosine (□) or 1 mM inosine (▲; not significantly different from zero, *P* > 0.05). (D) Transport of 0.5 μM [^3^H]-uridine by B48 cells transfected with LmajNT2 (●), which was inhibited 95.2% by a mixture of 250 μM adenosine and 250 μM uracil (□; rate not significantly different from zero, F-test)). Inhibition by 1 mM uridine is also indicated (▲). Unit for transport was pmol(10^7^ cells)^−1^ in all frames; symbols represent the average of triplicate determinations in a single representative experiment, and error bars represent SEM.

NT2 has, to date, only been investigated in *Leishmania donovani*, and was found to be an adenosine-insensitive inosine/guanosine (oxopurine nucleoside) transporter (Carter et al., 2000). We thus took advantage of our *T. brucei* B48 expression system, in which all endogenous inosine transport is highly sensitive to inhibition by adenosine, to determine whether NT2 of *L. major* and *L. mexicana* are likewise able to transport inosine in an adenosine-insensitive way. B48 cells transfected with either LmajNT2 (Fig. 9A) or LmexNT2 (Fig. 9B) displayed an [^3^H]-inosine transport activity which was only partly inhibited by 250 μM adenosine. Since adenosine, at these high concentrations, inhibits all inosine transport ([^3^H-]-inosine at 50 nM) in untransfected B48 cells (Fig. 7B), the remaining uptake rate in the presence of saturating concentrations of adenosine, must be mediated by the heterologously expressed *Leishmania* transporter. From this experiment it is evident that the *Leishmania* NT2 transporters are not sensitive to inhibition by 250 μM adenosine. This was also directly shown using *L. mexicana* promastigotes, where [^3^H]-inosine transport was not inhibited at all by 1 mM adenosine (Fig. 9C). Furthermore, it could be established that uridine is not a substrate for LmajNT2 either, since transport of 0.5 μM [^3^H]-uridine in B48 expressing this transporter was fully inhibited by a mixture of 250 μM adenosine plus 250 μM uracil (Fig. 9D), which inhibits all endogenous *T. brucei* uridine transport (Fig. 7A) but does not inhibit LmajNT2.

We thus conclude that the *L. major* and *L. mexicana* NT2 transporters are indeed equivalent to the previously characterized *L. donovani* NT2 transporter, in that they are efficient transporters of low concentrations of inosine and insensitive to the *Leishmania* NT1 substrates adenosine and uridine.

### 3.4 Sensitivity of *Leishmania* species to pyrimidine nucleoside and nucleobase analogs

As pyrimidine nucleosides were salvaged much better by the *Leishmania* NT1 transporters than by the nucleoside transporters of *T. brucei* (Gudin et al., 2006, Ali et al., 2013a), we decided to assess the antileishmanial effects of a selection of potentially cytotoxic pyrimidine analogs. The only pyrimidine analogs tested that showed activity against promastigotes were 5-fluorouracil (5-FU), 5-fluoro-2′-deoxyuridine (5F-2′dUrd) and 5-fluoro-2′-deoxycytidine (5F-2′dCtd); these analogs displayed EC_50_ values at the low-to-mid micromolar level (Table 3), with the thymidine analog 5F-2′dUrd showing the most potent activity at ∼1.5 μM against both *Leishmania* species. Interestingly, the uridine analog 5′-deoxyuridine showed much lower activity against *L. mexicana* promastigotes, with an EC_50_ value of 461 ± 80 μM, which shows that all the uridine analogs had poor antileishmanial activity whereas the 2′-deoxyuridine analog 5F-2′dUrd, masquerading as a thymidine analog, was several orders of magnitude more effective.

**Table 3:** Sensitivity of 5-FU and 5F2′dURes clones to fluorinated pyrimidines.

	*L. mexicana*					*L. major*				
	WT	Lmex5FURes		Lmex5F2′dURes		WT	Lmaj5FURes		Lmaj5F2′dURes	
	EC_50_	EC_50_	RF	EC_50_	RF	EC_50_	EC_50_	RF	EC_50_	RF
5-FU	9.3 ± 0.6	1374 ± 123	147	1774 ± 301	190	8.5 ± 0.6	150 ± 5	17	12 ± 1.0	1.3
5F-2′dUrd	1.4 ± 0.06	1.5 ± 0.2	1.07	>5000	>3500	1.7 ± 0.1	6.1 ± 0.5	3.6	381 ± 83	224
5F-2′dCtd	17.3 ± 1.8	24 ± 0.9	1.4	>5000	>290	38 ± 1.7	17 ± 1.8	0.4	3870 ± 621	101
5F-Urd	>5000	>5000	1	>5000	1	18 ± 1.6	110 ± 17	6.1	1230 ± 340	68
5′dUrd	461 ± 80	ND	–	ND	–	525 ± 46	ND	–	ND	–
pentamidine	4.6 ± 0.2	5.0 ± 0.6	1.1	ND	–	3.3 ± 0.2	ND	–	ND	–
diminazene	7.4 ± 0.3	7.2 ± 1.4	0.97	13.9 ± 1.9	1.8	9.8 ± 0.3	10 ± 1.6	1.02	24.6 ± 1.5	2.5

All EC_50_ values were obtained using the Alamar blue assay and are given in μM. WT = wild-type sensitive control strain. Resistance Factor = IC_50_ (resistant clone)/IC_50_ (WT); n ≥ 4. ND, not done. The following pyrimidine analogs were ineffective against promastigotes of either species (EC_50_ > 5000 μM): 5-fluoroorotic acid, 5-chloro-2′-deoxyuridine, 6-azauracil, 5′-deoxy-5′-fluorouridine, 2′,3′-dideoxyuridine, 3′-deoxyuridine, 2-thiouridine, 4-thiouridine, 5-chlorouridine, 5-iodouridine, 5-iodo-2′-deoxyuridine, 5-bromouracil, 5-bromouridine, 5-bromo-2′-deoxyuridine, 5-fluorocytosine, 5-fluorocytidine.

The effects of the fluorinated pyrimidine analogs was similar against *L. mexicana* and *L. major* promastigotes, an important observation with respect to any drug development, with the exception of 5-fluorouridine, which displayed no activity against *L. mexicana* at concentrations up to 5 mM, but killed promastigotes of *L. major* at concentrations below 20 μM (Table 3; Supplemental Fig. S4). This may indicate that, unlike *L. mexicana*, *L. major* is able to metabolically incorporate 5F-uridine, probably through a uridine or thymidine phosphorylase, which converts uridine and/or thymidine to uracil, which is subsequently phosphoribosylated to UMP by uracil phosphoribosyl transferase (Wilson et al., 2012). A list of pyrimidine analogs without significant activity against *Leishmania* promastigotes (EC_50_ > 5 mM) is given in the legend to Table 3. From this, it can be concluded that halogenation at position 5, other than with fluorine, results in effective analogs (Cl/Br/I are too large), that both 2-thio and 4-thio uridine are ineffective (presumably not substrates for thymidine kinase or for the UP/UPRT route); 5F-Ctd is ineffective (deamination would result in 5F-Urd); 3′-deoxy analogs are ineffective, including 2′,3′-dideoxyuridine (presumably not a substrate of thymidine kinase).

### 3.5 Development and characterization of 5-FU and 5F-2′dUrd-resistant *Leishmania* clones

As described in the Methods section, promastigotes of *L. mexicana* and *L. major* were adapted by *in vitro* exposure to 5-FU and 5F-2′dUrd. It was noted that *Leishmania* cells were adapted to 5F-2′dUrd more quickly than to 5-FU; while *Leishmania* cells become insensitive to high concentrations of 5F-2′dUrd in a few months, the resistance induction to 5-FU required approximately one year (Supplemental Fig. S2). Clonal lines were generated from each strain that displayed resistance to high concentrations of 5-FU or 5F-2′dUrd. 5-FU-adapted clones from *L. mexicana* and *L. major* were abbreviated Lmex-5FURes and Lmaj-5FURes, whereas cells adapted to 5F-2′dUrd were called Lmex-5F2′dURes and Lmaj-5F2′dURes, respectively.

The anti-leishmanial activities and cross-resistance patterns of selected fluorinated pyrimidine analogs were investigated for each adapted cell line, in parallel with the parental wild-type control strains (Table 3). It can be seen that Lmex-5FURes cells displayed high levels of resistance to 5-FU, but retained the same sensitivity to 5F-2′dUrd. On the other hand, Lmex-5F2′dURes were highly cross-resistant to 5-FU and to 5F-2′dCtd. As far as the *L. major* cell lines are concerned, the 5-FU adapted cell line was only slightly cross-resistant with 5F-2′dUrd and, if anything, slightly more sensitive to 5F-2′dCtd, whereas the Lmaj5F2′dURes clone was cross-resistant to 5F-2′dCtd but not to 5-FU.

**Fig. 10:**
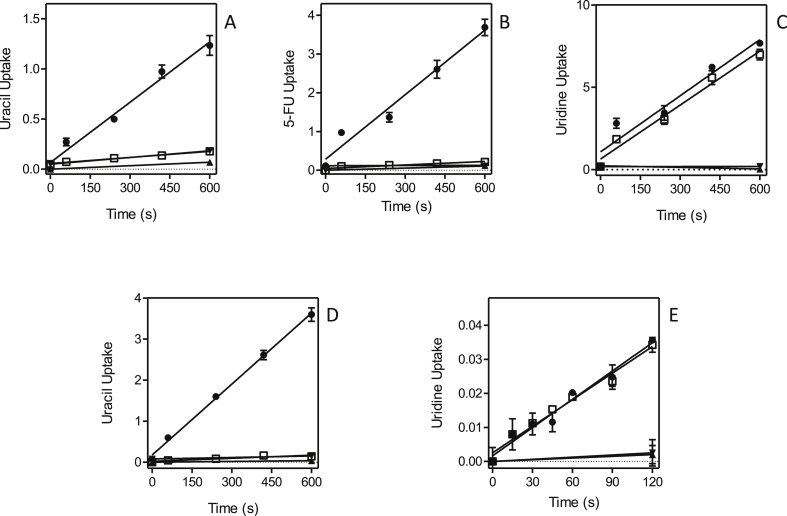
Pyrimidine transport by *Leishmania* clones adapted to 5-FU. (A) Uptake of 0.5 μM [^3^H]-uracil by Lmex5FURes (□) was 90.2% lower than for WT control cells (●), but still significantly different from zero (*P* = 0.0005, F-test). ▲,▼: WT and Lmex5FURes, respectively, in the presence of 1 mM uracil. (B) Like panel A, but with 0.5 μM [^3^H]-5-fluorouracil, which was 94.5% lower in Lmex5FURes than in WT cells, yet significantly non-zero (*P* = 0.024). (C) Like panel A, but with 0.5 μM [^3^H]-uridine, which was only 4.0% lower in Lmex5FURes than in WT cells (*P* > 0.05). (D) Uptake of 0.5 μM [^3^H]-uracil by Lmaj5FURes (□) was 95.7% lower than for WT control cells (●), but still significantly different from zero (*P* = 0.027, F-test). ▲,▼: WT and Lmaj5FURes, respectively, in the presence of 1 mM uracil. (E) Like panel D, but with 0.5 μM [^3^H]-uridine, which was statistically identical in Lmaj5FURes and WT cells (*P* > 0.05). Unit for transport was pmol(10^7^ cells)^−1^ in all frames; symbols represent the average of triplicate determinations in a single representative experiment, and error bars represent SEM.

**Fig. 11:**
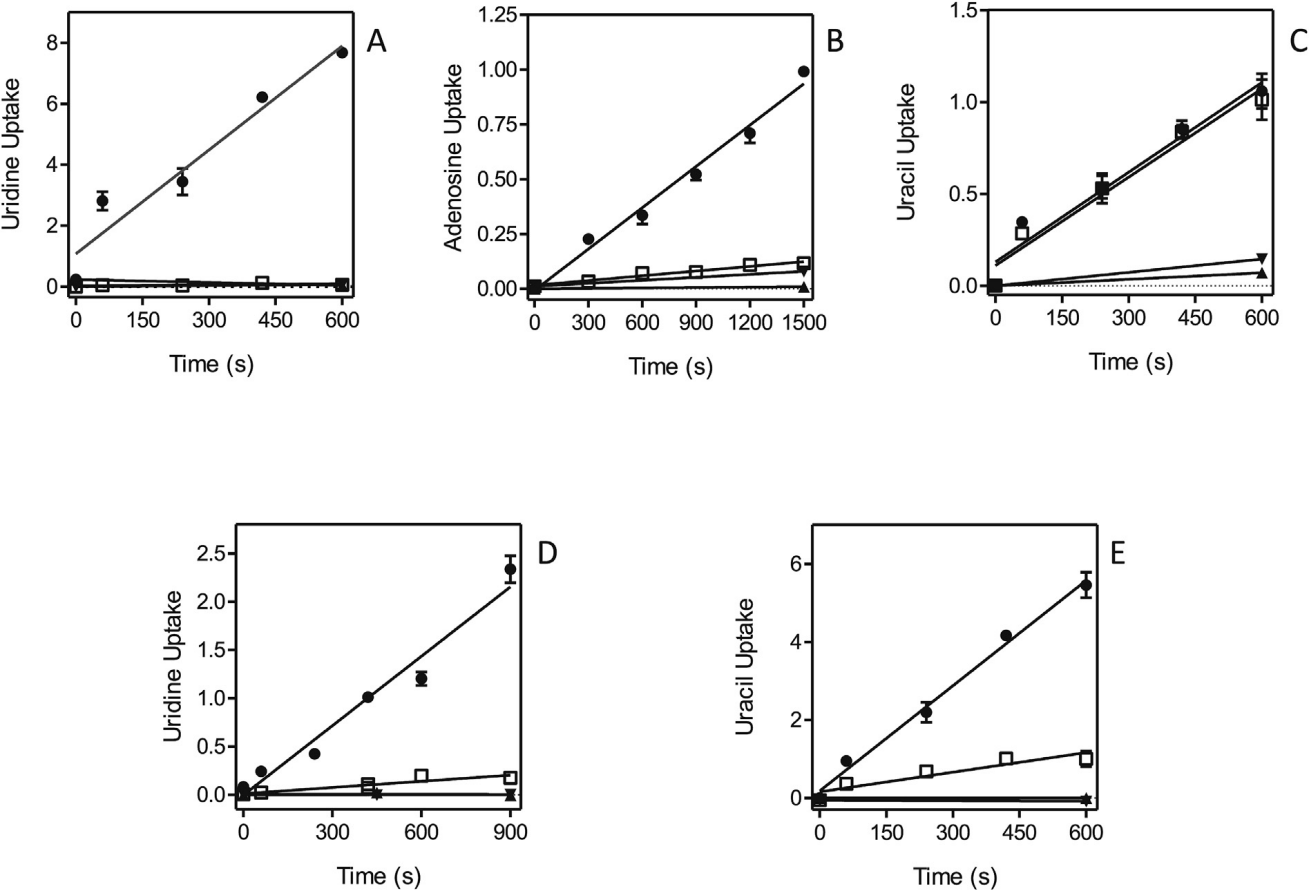
Pyrimidine transport by *Leishmania* clones adapted to 5-F2′dUrd. (A) Uptake of 0.5 μM [^3^H]-uridine in Lmex5F2′dURes cells (□) was 99% reduced compared to WT control cells (●) and not significantly different from zero (F-test, *P* = 0.27). ▲,▼: WT and Lmex5F2′dURes, respectively, in the presence of 1 mM uridine. (B) Uptake of 0.1 μM [^3^H]-adenosine in Lmex5F2′dURes (□) was 89% lower than in WT control cells, but significantly different from zero (*P* = 0.0005). ▲,▼: WT and Lmex5F2′dURes, respectively, in the presence of 1 mM adenosine. (C) Like frame A but measuring the uptake of 0.25 μM [^3^H]-uracil, which was not significantly different in Lmex5F2′dURes (□) and WT (●) cells. ▲,▼: WT and Lmex5F2′dURes, respectively, in the presence of 1 mM uracil. (D) Uptake of 0.25 μM [^3^H]-uridine was 90.1% lower in Lmaj5F2′dURes (□) than in WT control cells (●), and significantly different from zero (*P* = 0.007). ▲,▼: WT and Lmaj5F2′dURes, respectively, in the presence of 1 mM uridine. (E) Uptake of 0.5 μM [^3^H]-uracil was 81.4% lower in Lmaj5F2′dURes (□) than in WT control cells (●), and significantly different from zero (*P* = 0.025). ▲,▼: WT and Lmaj5F2′dURes, respectively, in the presence of 1 mM uracil. Unit for transport was pmol(10^7^ cells)^−1^ in all frames; symbols represent the average of triplicate determinations in a single representative experiment, and error bars represent SEM.

Part of the resistance phenotype could be the result of changes to the uptake efficiency of the fluorinated pyrimidines and this was therefore investigated next. Fig. 10 shows that in both of the 5-FU-adapted clones, Lmex-5FURes and Lmaj-5FURes, 0.5 μM [^3^H]-uracil transport was virtually absent, in contrast to solid levels of uptake in the control wild-type cells. This is a strong indication that resistance was the result of loss of the uracil transporter, a conclusion further strengthened by the absence of [^3^H]-5-FU transport in Lmex-5FURes but not wild-type cells (Fig. 10B). In contrast, the uptake of [^3^H]-uridine was not affected in either of the two resistant cell lines (Fig. 10C,E), which shows that the adaptation to 5-FU specifically involves the loss of the *Leishmania* U1 transporters and does not affect the activity of NT1. Similarly, the adaptation to 5F-2′dUrd involved the complete or near-complete abolition of uridine and adenosine uptake (Fig. 11A,B,D). In the adapted cell line Lmex5F2′dURes, uracil uptake was not affected at all (Fig. 11C), whereas it was clearly reduced in Lmaj5F2′dURes (Fig. 11E), although not as much as uridine uptake was in these cells (Fig. 11D). From these results it is clear that the main adaptation to 5F-2′dUrd in both *Leishmania* species was the drastic reduction of NT1-mediated nucleoside transport. In addition, there was a large reduction in LmajUU1 activity, which presumably further reduced uptake of 5F-2′dUrd.

### 3.6 Metabolomic investigation of the mechanism of action of fluorinated pyrimidines against *Leishmania*

The mechanism of action of the fluorinated pyrimidines was investigated using a metabolomics approach, where promastigotes of *L. mexicana* and *L. major* were incubated for 8 h with 100 μM of either 5-FU, 5F-2′dUrd or 5F-Urd prior to metabolite extraction and mass spectrometric analysis as described (Ali et al., 2013a, Alkhaldi et al., 2015). These conditions were chosen to allow for significant metabolite accumulation while not affecting cell viability or growth rate, which could broadly affect metabolite levels.

#### 3.6.1 5-fluorouracil

**Fig. 12:**
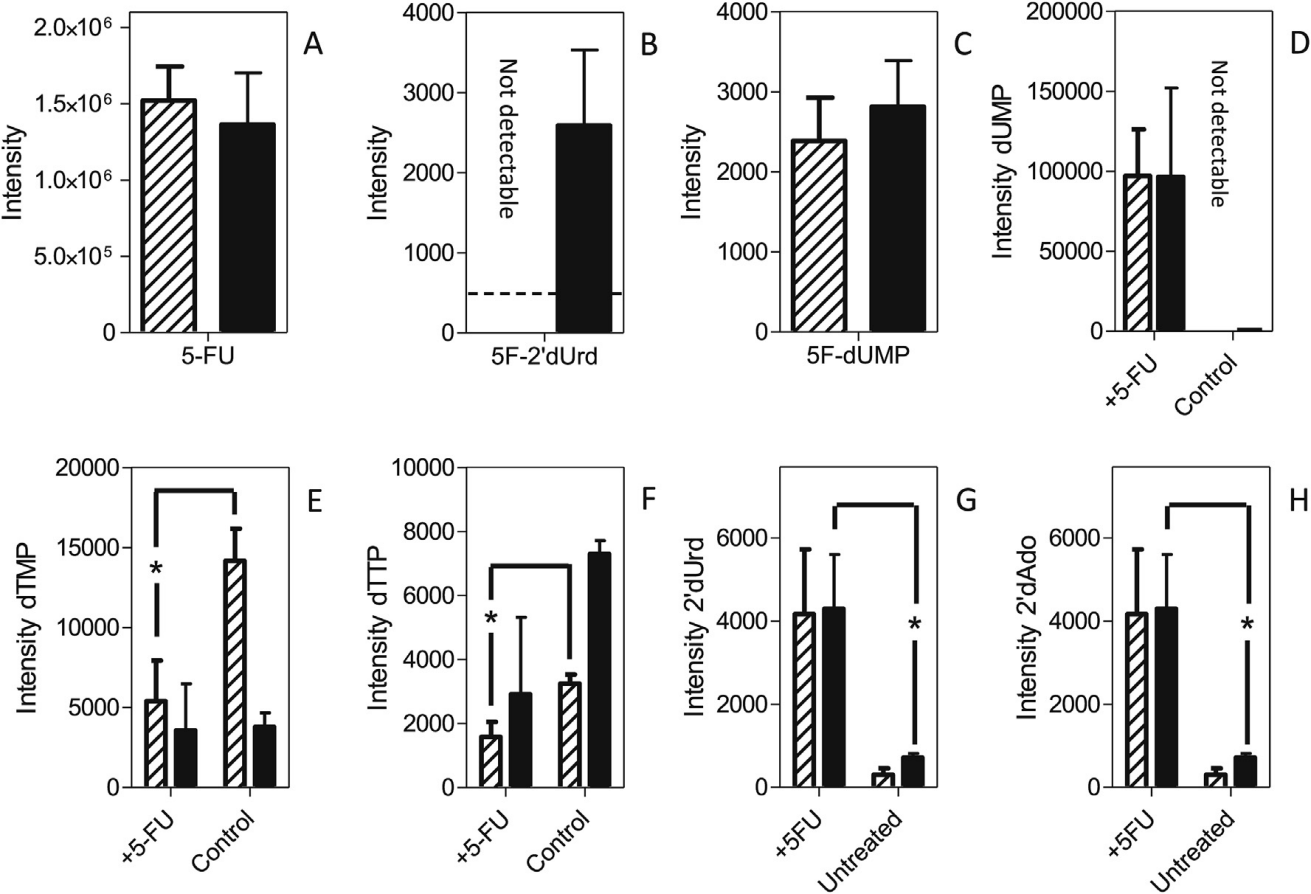
Metabolomic analysis of *L. mexicana* and *L. major* promastigotes treated for 8 h with 100 μM 5-FU. Hatched bars represent *L. mexicana*; solid bars, *L. major*. Panels A–C represent the relative intensity, in arbitrary units, of 5-FU (A), 5F-2′dUrd (B), or 5F-dUMP (C) in 5-FU-treated promastigotes. Panels D–H represent relative abundance of the indicated metabolites in 5-FU-treated promastigotes and untreated control cells: dUMP (D); dTMP (E); dTTP (F); 2′dUrd (G) and 2′dAdo (H). The results are the mean and SEM of triplicate determinations; *, *P* < 0.05, unpaired Student's t-test. Not detectable: the metabolite peak could not be identified in the sample with the requisite certainty. The dashed line in Frame B indicates the detection limit, set at 500 units.

As expected from the characterization of the *Leishmania* uracil transport activities, promastigotes of *L. mexicana* and *L. major* treated with 100 μM 5-FU contained a considerable amount of intracellular 5-FU (Fig. 12A). Neither fresh medium nor intracellular untreated controls showed any fluorinated pyrimidines, confirming that these accurate mass LC-MS peaks are specific for the fluorinated pyrimidines. 5F-2′dUrd was detected in *L. major*, showing 5-FU to be deoxyribosylated by a thymidine phosphorylase (5-FU presumably functioning as a thymine analog), but for *L. mexicana* the level was below the level at which it could confidently be detected with this metabolomics workflow (Creek et al., 2012) (Fig. 12B). However, *L. mexicana* also appears to generate 5F-2′dUrd from 5-FU, as almost identical levels of 5F-dUMP were observed in both *Leishmania* species (Fig. 12C). This shows that 5F-2′dUrd is a substrate for *Leishmania* thymidine kinase, as reported previously by Timm and coworkers (Timm et al., 2015), but no 5F-dUDP or 5F-dUTP were detected in any of the samples. Another common aspect between the *Leishmania* promastigotes was that neither 5-fluorouridine nor fluorinated uridine ribonucleotides (5F-UMP, 5F-UDP and 5F-UTP) were observed in extracted 5-FU-treated promastigotes, in complete contrast to 5-FU metabolism in *T. brucei*, where numerous such metabolites were found after exposure of bloodstream forms to 5-FU (Ali et al., 2013a, Ali et al., 2013b). We conclude that 5-FU is not a substrate for *Leishmania* uracil phosphoribosyl transferase (UPRT), and that 5F-2′dUrd is its only direct metabolite, analogous to the deoxyribosylation of thymine to thymidine, by uridine phosphorylase and/or by a separate thymidine phosphorylase activity. This also implies that the detected deoxy 5-FU nucleotides cannot have been derived from 5F-UDP via ribonucleotide reductase.

The main metabolic change in 5-FU-treated cells was a change in the level of deoxy-pyrimidine nucleotides. The intracellular levels of dUMP in 5-FU-treated promastigotes of both species were very strongly increased compared with respective untreated controls. Indeed the dUMP level in untreated control cells was very low, particularly in *L. mexicana*, where the level in untreated cells was below automatic detection, precluding statistical analysis in this instance (Fig. 12D). In addition, 5-FU caused a reduction in the intensity of the dTMP and dTTP peaks in *L. mexicana* promastigotes (*P* = 0.05 and *P* = 0.03, respectively, for *L. mexicana* compared with respective untreated control) (Fig. 12E and F). It should be noted that in treated *L. major* promastigotes the reduced level of deoxythymidine nucleotides was not significant, although a similarly reduced dTTP level was observed in two out of three replicates. The reduction in thymidine nucleotides could conceivably be the result of a reduced rate of thymidine uptake; although the HOMEM medium in which the experiment was performed does not contain any thymidine, the added fetal bovine serum would be expected to contain a small amount of thymidine. However, we found no significant difference in the free cellular thymidine levels between 5-FU-treated and untreated cells. The reduction in thymidine nucleotides in *Leishmania* species is thus probably due to the inhibition of thymidine kinase and thymidine synthase by 5-FU or, more likely, its metabolites 5F-2′dUrd and 5F-dUMP, which are analogous to thymidine and TMP, respectively. It is the inhibition of dihydrofolate reductase-thymidine synthase (DHFR-TS), evidenced by the massive build-up of dUMP in the cell, that may be the most relevant as it is an essential enzyme in *Leishmania* (Titus et al., 1995), and the target for antifolates such as methotrexate (Vickers and Beverley, 2011). It is further probable that 5F-dUMP and/or 5F-2′dUrd inhibited thymidylate kinase, another key metabolic enzyme (Thiel et al., 2008), explaining the reduced levels of TTP.

Other changes that occurred in both *L. mexicana* and *L. major* treated with 5-FU were an elevation of the levels of 2′deoxyuridine (Fig. 12G; *P* = 0.06 and 0.05, respectively) and 2′deoxyadenosine (Fig. 12H; P < 0.05 for *L. major*) compared to untreated controls. We also observed large increases in the intensity of 2′deoxynucleotides (dCMP, dCDP, dCTP, dAMP) relative to respective untreated controls (Table 4). It must be noted that the level of 2′deoxycytidine and 2′deoxyadenosine nucleotides were barely detected in *Leishmania* untreated controls; dATP was undetectable in both treated and untreated cells; therefore, no statistical analysis can be performed here and onwards. The increase in 2′-deoxynucleosides and -nucleotides points to an effect on ribonucleotide reductase, which, in *T. brucei*, is allosterically regulated by deoxynucleotides (Hofer et al., 1998).

**Table 4:** Peak intensities of deoxynucleosides and nucleotides after metabolomic analysis.

	*L. major*				*L. mexicana*			
	Untreated	+5-FU	+5F2′dUrd	+5FUrd	untreated	+5-FU	+5F2′dUrd	+5FUrd
dCMP	not detected	5130	not detected	6744 ± 3500	not detected	5636 ± 3288	6006 ± 3089	12757 ± 1038^∗∗^
dCDP	not detected	6197 ± 1588	6617 ± 188^∗∗∗^	6904 ± 384^∗∗^	not detected	4803 ± 433^∗∗^	4451 ± 1188^∗^	10404 ± 1470^∗^
dCTP	995 ± 109	4541 ± 565	7036 ± 1684	5477 ± 1350^∗^	not detected	2913 ± 1346	1569 ± 244	2338 ± 428^∗^
dAdo	2992 ± 173	6996 ± 2423	14181 ± 3275	11818 ± 839^∗∗^	2585 ± 811	6476 ± 2403	5412 ± 1699	8045 ± 4537
dAMP	6192 ± 910	19379 ± 3774	14740 ± 2815	18001 ± 2307^∗^	10660 ± 2897	20366 ± 5358	30100 10459	21978 ± 1784^∗∗^
dADP	not detected	not detected	6493 ± 3261	6482 ± 3260	1458	8948 ± 461^∗∗^	8367 ± 1844^∗^	9404 ± 1389^∗^
dUrd	712 ± 105	5177 ± 1445	7615 ± 1236^∗^	8145 ± 1684^∗^	304 ± 155	4166 ± 1560	5414 ± 717^∗^	6439 ± 456^∗^

**Fig. 13:**
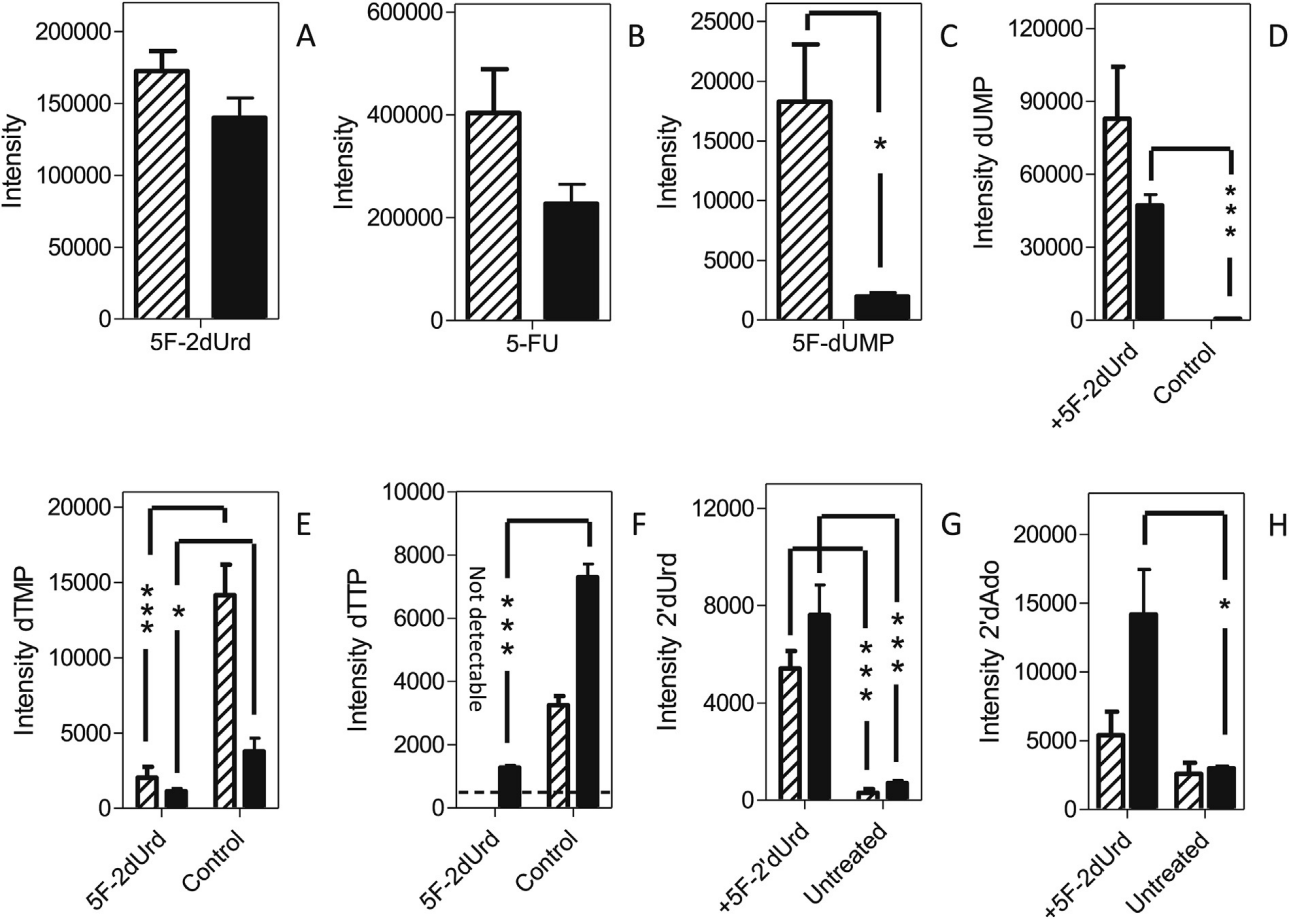
Metabolomic analysis of *L. mexicana* and *L. major* promastigotes treated for 8 h with 100 μM 5F-2′dUrd. Hatched bars represent *L. mexicana*; solid bars, *L. major*. Panels A–C represent the relative intensity, in arbitrary units, of 5F-2′dUrd (A), 5-FU (B), or 5F-dUMP (C) in 5F-2′dUrd -treated promastigotes. Panels D–H represent relative abundance of the indicated metabolites in 5F-2′dUrd -treated promastigotes and untreated control cells: dUMP (D); dTMP (E); dTTP (F); 2′dUrd (G) and 2′dAdo (H). The results are the mean and SEM of triplicate determinations; *, *P* < 0.05; ***, *P* < 0.001 (unpaired Student's t-test). The dashed line in Frame F indicates the detection limit, set at 500 units.

**Fig. 14:**
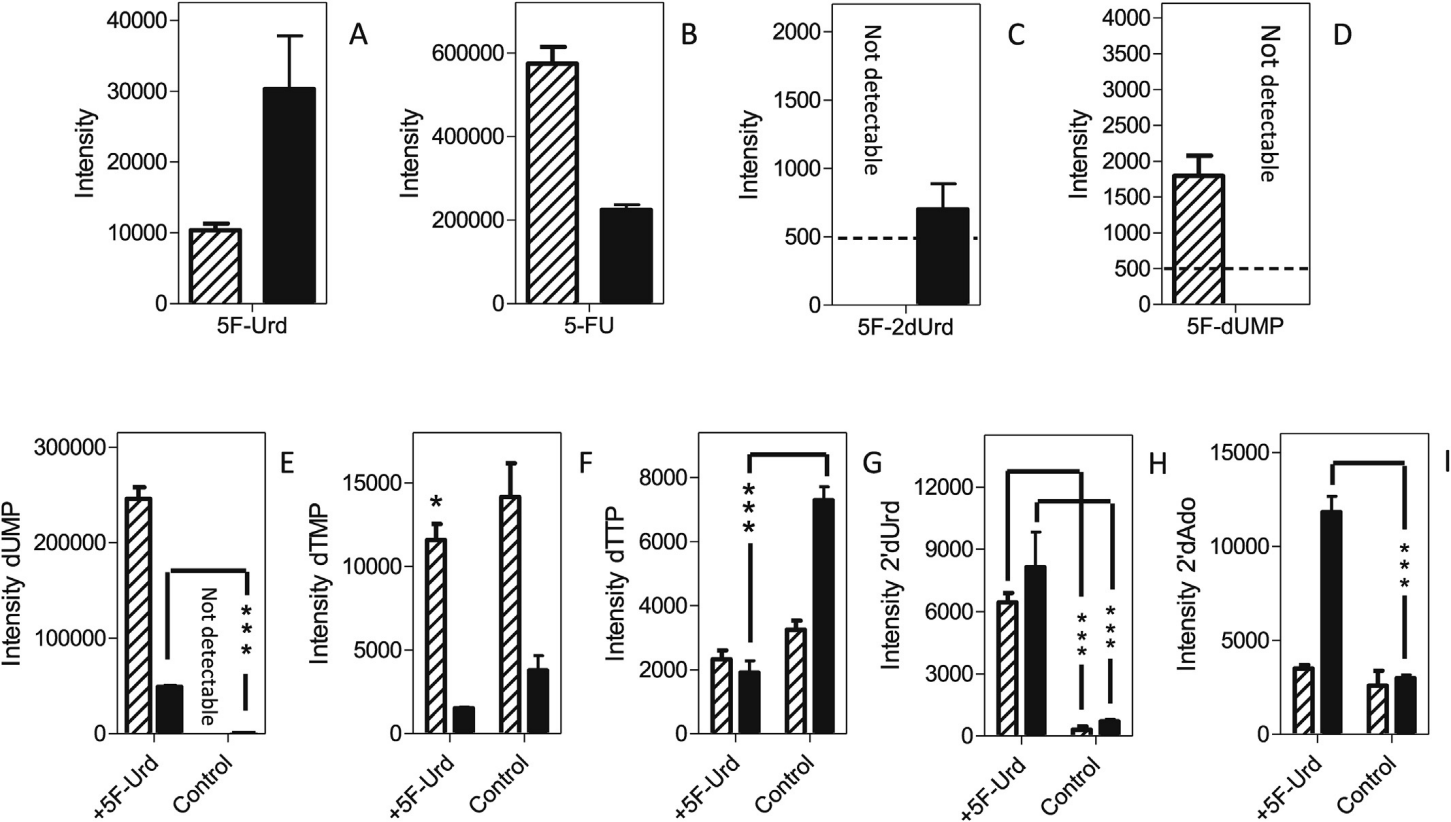
Metabolomic analysis of *L. mexicana* and *L. major* promastigotes treated for 8 h with 100 μM 5F-Urd. Hatched bars represent *L. mexicana*; solid bars, *L. major*. Panels A–C represent the relative intensity, in arbitrary units, of 5F-Urd (A), 5-FU (B), 5F-2′dUrd (C), or 5F-dUMP (D) in 5F-Urd-treated promastigotes. Panels E–I represent relative abundance of the indicated metabolites in 5F-Urd-treated promastigotes and untreated control cells: dUMP (E); dTMP (F); dTTP (G); 2′dUrd (H), 2′dAdo (I). The results are the mean and SEM of triplicate determinations; *, *P* < 0.05; ***, *P* < 0.001 (unpaired Student's t-test). The dashed line in Frames C and D indicates the detection limit, set at 500 units.

Average (n = 3) and SEM of peak intensities in arbitrary units as generated by the Orbitrap mass spectrometer. Statistical significance relative to untreated controls was determined using an unpaired Student's t-test. *, *P* < 0.05; **, *P* < 0.01; ***, *P* < 0.001. Not detected implies that the signal was below 500 arbitrary units. The peak intensities of deoxy nucleotides dTMP, dTTP, 2′dUrd and dUMP have been depicted in Fig. 12, Fig. 13, Fig. 14.

#### 3.6.2 5-Fluoro-2′deoxyuridine

5F-2′dUrd appears to be taken up robustly by promastigotes of both *Leishmania* species, as intense peaks were detected in both extracts after incubation with 100 μM of 5F-2′dUrd for 8 h (Fig. 13A). In addition, high levels of 5-FU were found after the treatments (Fig. 13B), showing that the uridine phosphorylase/thymidine phosphorylase catalyzes the reversible conversion of 5-FU to 5F-2′dUrd. As described above for the incubation with 5-FU, no 5F-Urd was detected. 5F-dUMP was detected and the cellular concentration was significantly higher in *L. mexicana* than in *L. major* (Fig. 13C), the difference likely being the result of a more rapid conversion of 5F-2′dUrd to 5F-dUMP in *L. mexicana*, which is also consistent with the non-detection (depletion) of 5F-2′dUrd in 5-FU-treated *L. mexicana* (Fig. 12B), where 5F-2′dUrd was present at a much lower level as it needed to be generated first from 5-FU. The higher level of 5F-2′dUrd in *L. mexicana* also caused a stronger or earlier inhibition of TS and a correspondingly higher level of dUMP in the cells (Fig. 13D), although the treatment caused strong depletions of dTMP and dTTP in both of the *Leishmania* species (Fig. 13E and F); indeed, in 5F-2′dUrd-treated *L. mexicana*, dTTP could no longer be identified in any of the replicates.

As seen above with 5-FU, we also observed strong increases in the level of 2′deoxyuridine of both *L. mexicana* and *L. major* (Fig. 13G; *P* < 0.001), and an increase in the 2′deoxyadenosine intensity (Fig. 13H; not significant in *L. mexicana*, *P* = 0.027 in *L. major*). It was further observed that treatment with 5F-2′dUrd, like 5-FU, lead to similar increases in the levels of 2′deoxycytidine nucleotides (dCMP, dCDP, dCTP) and 2′deoxyadenosine nucleotides (dAMP, dADP) (Table 4).

#### 3.6.3 5-Fluorouridine

5F-Uridine was clearly taken up by both species as it was easily detectable intracellularly (Fig. 14A), although it was not observed in cells treated with 5-FU or 5F-2′dUrd. In both species, by far the highest intensity peak of a fluorinated pyrimidine was 5-FU (Fig. 14B), indicating that 5F-Urd was a substrate for a uridine phosphorylase and/or thymidine phosphorylase although it is not generated by it to any detectable level from 5-FU, indicating that the reaction equilibrium is strongly towards the phosphorolysis of 5F-Urd. Relatively low intensity peaks for 5F-2′dUrd (*L. major*; Fig. 14C) and 5F-dUMP (*L. mexicana*; Fig. 14D) were also observed. The higher level of 5F-dUMP in *L. mexicana* was also observed after treatment with 5F-2′dUrd (Fig. 13D) and, together with the below detection level of 5F-2′dUrd in cells treated with either 5-FU or 5F-Urd (Fig. 12B and Fig. 14C, respectively), strongly suggest that 5F-2′dUrd is a better substrate for thymidine kinase in *L. mexicana* than in *L. major*, leading to a build-up of 5F-2′dUrd in *L. major* but not in *L. mexicana*.

As with the other treatments, very large increases in dUMP levels were observed (Fig. 14E), and the intensity of thymidine nucleotides was decreased (Fig. 14F and G), although the latter changes were relatively minor compared to 5-FU and 5F-2′dUrd treatment. Interestingly, the peak intensities for 5F-dUMP and dUMP were much higher in *L. mexicana* than in *L. major*, although *L. mexicana* was insensitive to 5F-Urd up to 5 mM (Table 3). This seems to indicate that, at least in *L. mexicana*, high levels of dUMP alone are not sufficient to cause cell death, and that the depletion of dTTP is a better marker of the antileishmanial activity of fluorinated pyrimidines. Both species showed a similar increase in 2′-dUrd peak intensity (Fig. 14H) but only *L. major* showed a significant increase in 2′-deoxyadenosine (Fig. 14I); however, both species contained significantly increased levels in deoxycytidine nucleotides, dAMP and dADP (Table 4).

## 4 Discussion

Nucleotide metabolism in protozoa is replete with promising drug targets, and nucleoside analogs have become key players in anticancer and anti-viral chemotherapy. In *Trypanosoma brucei*, purine and pyrimidine transporters have been studied in great detail, as have many enzymes of nucleotide metabolism (De Koning and Jarvis, 1998, De Koning et al., 2005, Al-Salabi et al., 2007, Berg et al., 2010a, Berg et al., 2010b, Munday et al., 2013). This information is now being translated into the rational design of nucleoside analogs, with efficient uptake through known transporters and well-understood metabolic activation steps, as potential agents against African trypanosomiasis (Berg et al., 2010a, Vodnala et al., 2013, Vodnala et al., 2016, Rodenko et al., 2015, Ranjbarian et al., 2017). Developing new drugs for the leishmaniases, with their multiple pathologies and many causative species, is at least as urgent; yet the current knowledge of *Leishmania* purine and pyrimidine transporters and metabolism lags behind that of African trypanosomes. In this study we attempt to address several of the urgent questions pertinent to the development of a successful nucleoside-analog therapy against leishmaniasis: (1) Are the nucleoside transporters in various clinically important *Leishmania* species similar enough to allow the efficient uptake of the same analogs? (2) Would the *Leishmania* nucleoside transporters, like their *T. brucei* counterparts, allow the uptake of modified nucleosides and if so, which modifications might be admissible? (3) How susceptible is the pyrimidine salvage system to pyrimidine analogs, including 5-halogenated pyrimidines that have been widely used in anti-cancer chemotherapy, and could these be repurposed? (4) How are such pyrimidine analogs metabolized in *Leishmania*, are there significant differences between species, and how does this compare to the same process in African trypanosomes?

Since more than 20 different *Leishmania* species contribute to the various clinical manifestations of leishmaniasis world-wide, it is virtually essential that any new treatments developed should be efficacious to at least the main pathogenic species involved, and this issue should be addressed at the very onset of a program, before major resources are invested in a strategy. In that context we address here whether nucleoside transport in *L. mexicana* and *L. major* are substantially different from each other, and from the *L. donovani* transporters reported earlier (see Introduction). The three ENT-family nucleoside transporters (NT1.1, NT1.2 and NT2) seem to be syntenically preserved throughout the genus. We cloned these genes from *L. mexicana* and *L. major*, and expressed them heterologously in the related trypanosomatid *T. brucei*. The results show that, as in *L. donovani* (Vasudevan et al., 1998, Carter et al., 2000), the NT1 transporters mediate the uptake of uridine and adenosine but not inosine, whereas the NT2 transporters facilitate inosine but not uridine uptake. Thus, we conclude that the organization of nucleoside transport is preserved in *Leishmania* species causing mainly visceral (*L. donovani*), ‘old world’ cutaneous (*L. major*) and diffuse cutaneous and muco-cutaneous ‘new world’ leishmaniasis (*L. mexicana*).

**Fig. 15:**
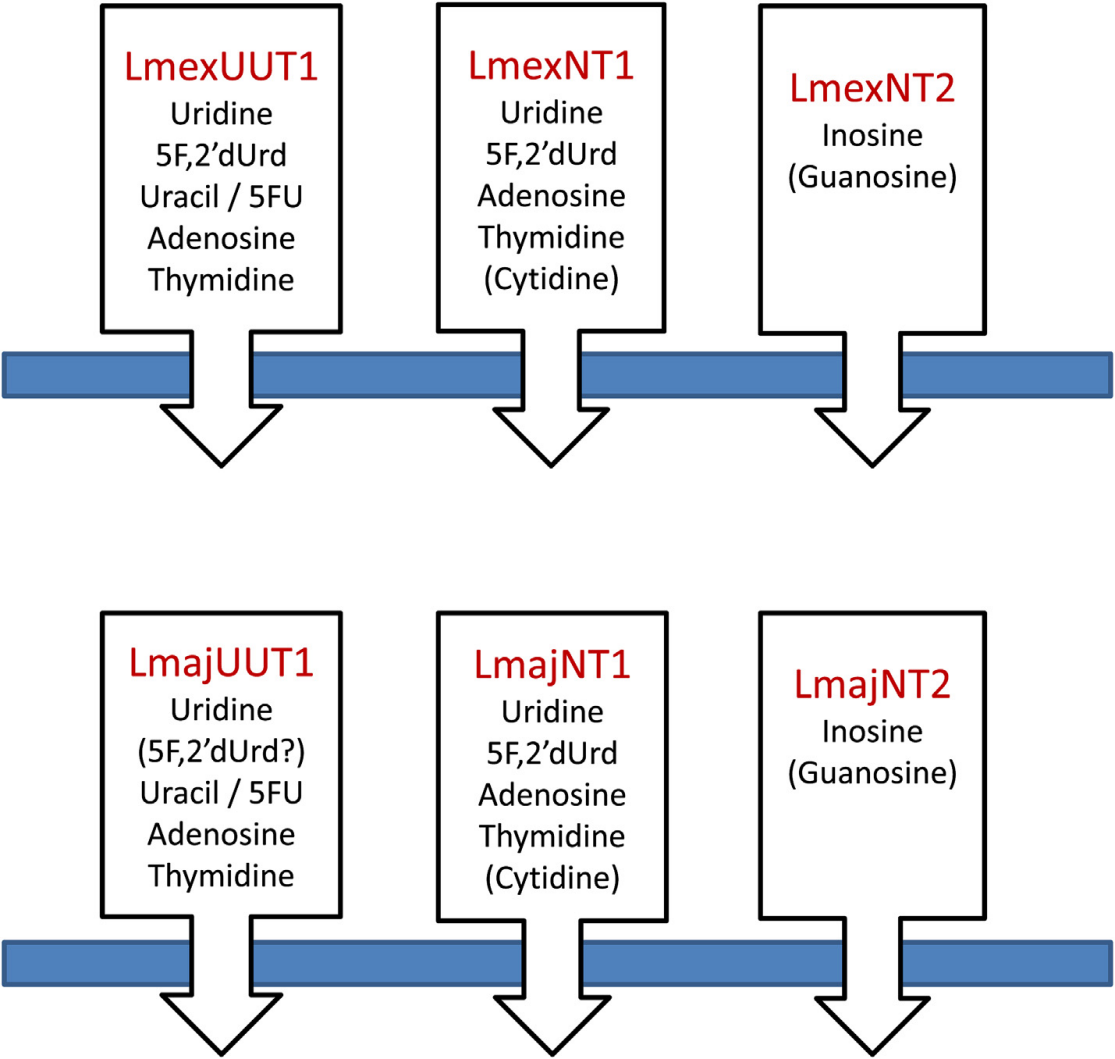
Schematic model of pyrimidine transport activities in *L. mexicana* (top panel) and *L. major* (lower panel). The blue bars represent the parasite plasma membranes, with the arrow heads pointing to intracellular space. Each box-arrow represents a transport activity, identified in bold red font, with substrates listed underneath; expected substrates that were not tested in this study or of low affinity are listed in brackets.

Functional studies with *L. donovani* promastigotes and individual *L. donovani* nucleoside transporters expressed in *Xenopus laevis* oocytes established that LdNT1.1 was a higher affinity transporter than LdNT1.2 (Vasudevan et al., 1998, Landfear, 2001), with NT1.1 displaying K_m_ values of ∼0.2 μM and 5 μM for adenosine and uridine, respectively, whereas the corresponding values for NT1.2 were ∼5 μM and 40 μM, respectively. It was further reported that expression of NT1.2 was very low in promastigotes, contributing relatively little to the nucleoside transport activity. These observations are entirely compatible with our careful analysis of nucleoside uptake in promastigotes of *L. major* and *L. mexicana*, as we present clear evidence for a higher affinity and a lower affinity adenosine-sensitive uridine uptake activity. In both cases, the higher affinity transport was sensitive to inhibition by uracil, and at least in the case of *L. mexicana*, it was able to transport this nucleobase, and we therefore named this activity *L. mexicana* uridine-uracil transporter 1 (LmexUUT1), rather than NT1.1. However, the UU1 transporters of *L. mexicana* and *L. major* clearly are high affinity adenosine/uridine transporters, with a secondary ability to transport uracil (in *L. mexicana* V_max_/K_m_ [^3^H]-uracil = 0.003, compared to 0.14, 1.38 and 0.055 for [^3^H]-thymidine, [^3^H]-adenosine and [^3^H]-uridine, respectively), and are thus nucleoside transporters rather than nucleobase transporters. For *L. donovani* it was never tested whether either of the NT1-type transporters might be sensitive to inhibition by uracil, but by extension it could be speculated that LdNT1.1 might be homologous to LmexUUT1 and LdNT1.2 the equivalent of LmexNT1; this remains to be established. LmexUUT1 was sensitive to both thymidine and adenosine, as shown above for the equivalent activity LmajUUT1. In *L. major*, it was possible to detect two separate thymidine transport activities with K_m_ values of 4.2 μM and 26.9 μM, respectively. Thus, all three *Leishmania* species express 2 uridine transporters, which are also transporters for adenosine and thymidine (Fig. 15). In addition, *L. major*, at least, is reported to express two purine nucleobase transporters, LmajNT3 and LmajNT4 (Sanchez et al., 2004, Ortiz et al., 2007), as well as a high affinity (K_m_ = 0.32 μM) uracil transporter, LmajU1 (Papageorgiou et al., 2005).

We next addressed whether these transporters might allow the accumulation of cytotoxic nucleosides in *Leishmania* parasites. Previous authors had already shown uptake of the purine nucleoside analogs 7-deaza-adenosine (tubercidin) by LdNT1.1 (Vasudevan et al., 1998, Landfear, 2001) and of Formycin B by LdNT2 (Carter et al., 2000), as well as inhibition by 6-thioguanosine at 100-fold excess over inosine as a substrate (Carter et al., 2000). Here we tested a number of pyrimidine nucleoside analogs for inhibition of LmajNT1 and found that 5F-Urd and 5F-2′dUrd, in particular, but also 4-thiouridine and 5F-5′dUrd were able to inhibit this transporter. Using these and other results, we were able to construct a model for pyrimidine nucleoside binding by LmajNT1, which should assist in the further selection of possible substrates for this carrier, much as it has done for some of the *T. brucei* transporters for which we constructed similar models (Lüscher et al., 2007b, Chollet et al., 2009, Vodnala et al., 2013, Rodenko et al., 2015).

We further tested a series of pyrimidine nucleoside analogs for activity against *L. mexicana* and *L. major* promastigotes, and found several things of interest. First, we found that of 21 analogs tested, 20 exhibited highly similar activities against both species, with 5F-Urd being the sole exception as *L. mexicana* was completely unaffected by it (although it was clearly taken up, being detected intracellularly by the metabolomic analysis). Indeed, we found that 4-thiouridine and 5F-5′dUrd (which like 5F-Urd are inhibitors of LmajNT1) had no effect on promastigotes even at millimolar levels. This highlights a key challenge to developing a rational nucleoside therapy to protozoa, i.e. analogs must be compatible both with the transporters that facilitate their entry into the cell, and with the metabolic enzymes that are required to activate these prodrugs. Nonetheless, 5-FU, 5F-2′dCtd, and in particular 5F-2′dUrd, showed activity against both *Leishmania* species in the low-micromolar range. In this the *Leishmania* promastigotes were surprisingly similar to bloodstream form *T. brucei*: 5-FU, 5F-2′dUrd and 5F-2′dCtd were much more active against the trypanosomes than any of the other halogenated pyrimidines tested against those parasites (Ali et al., 2013a). The main difference between the *Leishmania* species and *T. brucei*, with regards to sensitivity to pyrimidine analogs, is that *Leishmania* promastigotes are insensitive to 5F-orotic acid, to which the trypanosomes are susceptible. This can be understood in the context of a pyrimidine auxotrophic clone *L. donovani* being unable to grow in 100 μM orotate as sole pyrimidine source (French et al., 2011), being unable to incorporate this precursor of UMP (in contrast to *T. brucei* (Ali et al., 2013b)).

Fluorinated pyrimidines have previously been introduced as pyrimidine salvage inhibitors against *T. gondii* (Youn et al., 1990) and *L. amazonensis* (Katakura et al., 2004). Here we do not necessarily propose fluorinated pyrimidines as antileishmanial lead compounds; rather, this study is the first effort to evaluate the metabolism of pyrimidine anti-metabolites by *Leishmania* parasites, and the mechanism by which they might exert their antileishmanial effects. We were able to adapt the *Leishmania* promastigotes to high levels of resistance to fluorinated pyrimidines. The cell lines adapted to 5F-2′dUrd were cross-resistant to 5F-2′dCtd, as has been reported also for *T. brucei* (Ali et al., 2013a), which highlights that the deoxy nucleoside analogs likely have the same mechanism of action (5F-2′dCtd being converted to 5F-2′dUrd by cytidine deaminase) as well as the same transporters. We also show here that in both *Leishmania* species, the 5-FU adaptation was associated with a near-complete ablation of uracil and 5-FU uptake, whereas uptake of uridine was not significantly affected. Similarly, the 5F-2′dUrd-resistant strains had lost >90% of uridine (and adenosine) uptake capacity, whereas uracil uptake in Lmex-5F2′dURes was unaffected, but 80% lower in Lmaj-5F2′dURes. We conclude that loss of NT1-like transport activity can account for much of the resistance phenotype. It has previously been shown that the *Leishmania* nucleoside transporters are not essential proteins under standard *in vitro* or *in vivo* conditions (Liu et al., 2006).

**Fig. 16:**
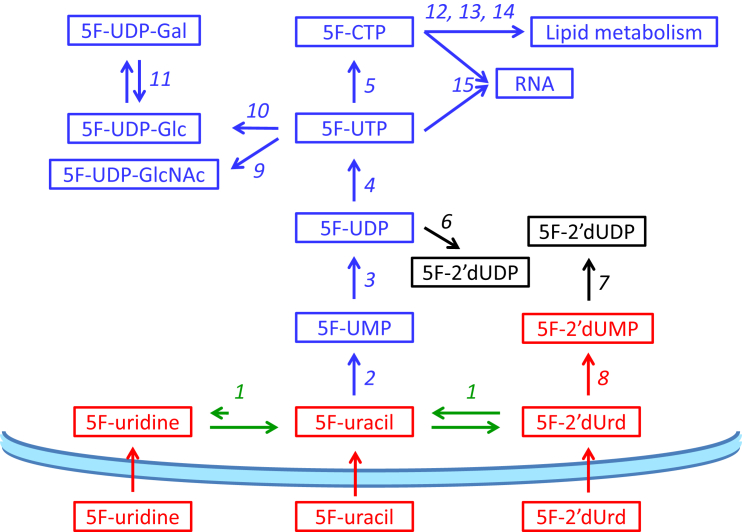
Schematic overview of fluoropyrimidine metabolism in *T. b. brucei* and *Leishmania* species. Red boxes and arrows represent metabolites and reactions, respectively, observed in both *T. brucei* and *Leishmania* species; Blue boxes and arrows are observed only in *T. brucei* and green arrows represent reactions observed only in the *Leishmania* species; black boxes and arrows indicate reactions that do not happen in either species. The curved blue bar schematically represents the plasma membrane of the parasites. Enzyme reactions are indicated with numbers as follows. 1, Uridine Phosphorylase and/or Thymidine Phosphorylase; 2, Uracil Phosphoribosyl transferase; 3, Nucleoside Diphosphatase; 4, Nucleoside Diphosphate Kinase; 5, Cytidine Triphosphate Synthase; 6, Ribonucleoside-diphosphate Reductase; 7, Thymidylate Kinase; 8, Thymidine Kinase; 9, UTP:N-acetyl-a-D-glucosamine-1-phosphate Uridylyltransferase; 10, UDP-Glucose Pyrophosphorylase; 11, UDP-Glucose Epimerase; 12, Phosphatidate Cytidylyltransferase; 13, Ethanolamine-phosphate Cytidylyltransferase; 14, Choline-phosphate Cytidylyltransferase; 15, RNA Polymerase.

Apart from the details of pyrimidine transport, the pyrimidine salvage pathways are believed to be identical in the various pathogenic kinetoplastids (Valente et al., 2016). However, we found that fluorinated pyrimidines were metabolized by strikingly different routes and caused different metabolic effects in *Leishmania* promastigotes when compared to those reported previously in *T. brucei*. In *T. brucei* (Fig. 16), 5-FU was a substrate for UPRT and incorporated into uridine ribonucleotides and RNA and other metabolites derived from uridine nucleotides, including CTP, lipid intermediates such as CDP-ethanolamine, and glycosylation intermediates such as UDP-hexoses/hexosamines (Ali et al., 2013a). In contrast, we show here that 5-FU is not a substrate for LmajUPRT or LmexUPRT as no fluorinated ribonucleotides could be detected, including 5F-UMP. Instead, the only primary metabolite of 5-FU detected in *Leishmania* promastigotes was 5F-2′dUrd, consistent with reports that *Leishmania* species contain “a thymidine phosphorylase activity” (LaFon et al., 1982). We propose that *Leishmania* UP might recognize both uridine and thymidine, and hence 5F,2′dUrd, a thymidine analogue; alternatively there could be separate phosphorylases for thymidine and uridine. The deoxyribosylation of 5-FU to 5F,2′dUrd requires a source of deoxyribose-1-phosphate, which is not normally highly abundant in the cell. We speculate that the elevated levels of 2′-deoxynucleotides detected in the metabolomic analyses, particularly dUMP, are the result of increased activity of ribonucleotide reductase and inhibition of thymidylate synthase, and that increased phosphorolysis of these excess deoxynucleotides may generate a source of deoxyribose-1-phosphate used in forming 2′dUrd from 5-FU. The 5F-2′dUrd is then activated by thymidine kinase (TK) to 5F-2′dUMP, as also reported in *T. brucei* (Ali et al., 2013a), which in turn inhibits thymidylate synthase (DHFR-TS) and gives rise to an accumulation of dUMP and further changes in deoxynucleotide metabolism, presumably through interference with the regulation of ribonucleotide reductase. Treatment with 5F-Urd followed the same metabolomic pattern, with the nucleoside first non-reversibly converted to 5-FU.

This model for the trypanocidal activity of fluorinated pyrimidines is compatible with observations made by Ritt and coworkers (Ritt et al., 2013), who sequenced a number of 5-FU-adapted *L. infantum*. They found the amplification of *DHFR-TS* as one main adaptation, in addition to point mutations in UP and TK, all consistent with our model. However, they also found point mutations in *LinfUPRT*, although we found no evidence of this enzyme being involved in 5-FU metabolism in *L. mexicana* and *L. major*. One explanation might simply be that 5-FU metabolism in *L. infantum* is different in this respect, but unlike UP and TK, the reintroduction of a wild-type copy of *LinfUPRT* only marginally reversed the resistance phenotype, and the authors point out that the same resistant strain had also lost all capacity for 5-fluorouracil uptake, implying that the UPRT mutation may have been an early mutation during the adaptation, conferring a marginal advantage that was superseded by a subsequent loss of transport activity (Ritt et al., 2013). Altogether we conclude that, like nucleoside transport, the mechanism of action of 5-fluoro pyrimidines is essentially conserved in multiple *Leishmania* species covering most of the spectrum of leishmaniasis pathologies. Finally, we propose that 5F-2′dUrd is a more promising therapeutic lead against leishmaniasis, as the nucleoside display a more potent anti-leishmanial activity, is efficiently quickly taken up the parasites, and does not require the UP activation step.

## Appendix A Supplementary data

Online data Online data

Supplementary Fig. s1–s5 Supplementary Fig. s1–s5

## References

[bib1] Al-Salabi M.I., De Koning H.P. (2005). Purine nucleobase transport in amastigotes of *Leishmania mexicana*: involvement in allopurinol uptake. Antimicrob. Agents Chemother..

[bib2] Al-Salabi M.I., Wallace L.J.M., De Koning H.P. (2003). A *Leishmania major* nucleobase transporter responsible for allopurinol uptake is a functional homologue of the *Trypanosoma brucei* H2 transporter. Mol. Pharmacol..

[bib3] Al-Salabi M.I., Wallace L.J.M., Lüscher A., Mäser P., Candlish D., Rodenko B., Gould M.K., Jabeen I., Ajith S.N., De Koning H.P. (2007). Molecular interactions underlying the unusually high affinity of a novel *Trypanosoma brucei* nucleoside transporter. Mol. Pharmacol..

[bib4] Ali J.A., Creek D.J., Burgess K., Allison H.C., Field M.C., Mäser P., De Koning H.P. (2013). Pyrimidine salvage in *Trypanosoma brucei* bloodstream forms and the trypanocidal action of halogenated pyrimidines. Mol. Pharmacol..

[bib5] Ali J.A., Tagoe D., Munday J.C., Donachie A., Morrison L.J., De Koning H.P. (2013). Pyrimidine biosynthesis is not an essential function for *Trypanosoma brucei* bloodstream forms. PLoS One.

[bib6] Alkhaldi A.A., Creek D.J., Ibrahim H., Kim D.H., Quashie N.B., Burgess K.E., Changtam C., Barrett M.P., Suksamrarn A., De Koning H.P. (2015). Potent trypanocidal curcumin analogs bearing a monoenone linker motif act on *Trypanosoma brucei* by forming an adduct with trypanothione. Mol. Pharmacol..

[bib7] Alvar J., Vélez I.D., Bern C., Herrero M., Desjeux P., Cano J., Jannin J., Den Boer M., WHO Leishmaniasis Control Team (2012). Leishmaniasis worldwide and global estimates of its incidence. PLoS One.

[bib8] Berg M., Kohl L., Van der Veken P., Joossens J., Al-Salabi M.I., Castagna V., Giannese F., Cos P., Versées W., Steyeart J., Grellier P., Haemers A., Degano M., Maes L., De Koning H.P., Augustyns K. (2010). Evaluation of nucleoside hydrolase inhibitors in the treatment of African trypanosomiasis. Antimicrob. Agents Chemother..

[bib9] Berg M., Van der Veken P., Goeminne A., Haemers A., Augustyns K. (2010). Inhibitors of the purine salvage pathway: a valuable approach for antiprotozoal chemotherapy?. Curr. Med. Chem..

[bib10] Biebinger S., Wirtz L.E., Lorenz P., Clayton C. (1997). Vectors for inducible expression of toxic gene products in bloodstream and procyclic *Trypanosoma brucei*. Mol. Biochem. Parasitol..

[bib11] Boitz J.M., Ullman B., Jardim A., Carter N.S. (2012). Purine salvage in *Leishmania*: complex or simple by design?. Trends Parasitol..

[bib12] Bridges D.J., Gould M.K., Nerima B., Mäser P., Burchmore R.J., De Koning H.P. (2007). Loss of the high-affinity pentamidine transporter is responsible for high levels of cross-resistance between arsenical and diamidine drugs in African trypanosomes. Mol. Pharmacol..

[bib13] Burchmore R., Wallace L.J.M., Candlish D., Al-Salabi M.I., Beal P., Barrett M.P., Baldwin S.A., De Koning H.P. (2003). Cloning, heterologous expression and in situ characterization of the first high affinity nucleobase transporter from a protozoan. J. Biol. Chem..

[bib14] Carter N.S., Drew M.E., Sanchez M., Vasudevan G., Landfear S.M., Ullman B. (2000). Cloning of a novel inosine-guanosine transporter gene from *Leishmania donovani* by functional rescue of a transport-deficient mutant. J. Biol. Chem..

[bib15] Carter N.S., Landfear S.M., Ullman B. (2001). Nucleoside transporters of parasitic protozoa. Trends Parasitol..

[bib16] Changtam C., De Koning H.P., Ibrahim H., Sajid S., Gould M.K., Suksamrarn A. (2010). Curcuminoid analogues with potent activity against *Trypanosoma* and *Leishmania* species. Eur. J. Med. Chem..

[bib17] Chiang C.W., Carter N., Sullivan W.J., Donald R.G.K., Roos D.S., Naguib F.N.M., el Kouni M.H., Ullman B., Wilson C.M. (1999). The adenosine transporter of Toxoplasma gondii; identification by insertional mutagenesis, cloning, and recombinant expression. J. Biol. Chem..

[bib18] Chollet C., Baliani A., Wong P.E., Barrett M.P., Gilbert I.H. (2009). Targeted delivery of compounds of *Trypanosoma brucei* using the melamine motif. Bioorg. Med. Chem..

[bib19] Creek D.J., Jankevics A., Burgess K.E., Breitling R., Barrett M.P. (2012). IDEOM: an Excel interface for analysis of LC-MS-based metabolomics data. Bioinformatics.

[bib20] Croft S.L., Olliaro P. (2011). Leishmaniasis chemotherapy – challenges and opportunities. Clin. Microbiol. Infect..

[bib21] De Koning H.P. (2007). Pyrimidine transporters of trypanosomes – a class apart?. Trends Parasitol..

[bib22] De Koning H.P., Al-Salabi M.I., Cohen A., Coombs G.H., Wastling J.M. (2003). Identification and characterisation of high affinity purine nucleoside and nucleobase transporters in *Toxoplasma gondii*. Int. J. Parasitol..

[bib23] De Koning H.P., Bridges D.J., Burchmore R.J. (2005). Purine and pyrimidine transport in pathogenic protozoa: from biology to therapy. FEMS Microbiol. Rev..

[bib24] De Koning H.P., Jarvis S.M. (1997). Hypoxanthine uptake through a purine-selective nucleobase transporter in *Trypanosoma brucei brucei* procyclics is driven by protonmotive force. Eur. J. Biochem..

[bib25] De Koning H.P., Jarvis S.M. (1998). A highly selective, high affinity transporter for uracil in *Trypanosoma brucei brucei*; evidence for proton-dependent transport. Biochem. Cell Biol..

[bib26] De Koning H.P., Jarvis S.M. (1999). Adenosine transporters in bloodstream forms of *T. b. brucei*: substrate recognition motifs and affinity for trypanocidal drugs. Mol. Pharmacol..

[bib27] De Koning H.P., Watson C.J., Jarvis S.M. (1998). Characterisation of a nucleoside/proton symporter in procyclic *Trypanosoma brucei brucei*. J. Biol. Chem..

[bib28] French J.B., Yates P.A., Soysa D.R., Boitz J.M., Carter N.S., Chang B., Ullman B., Ealick S.E. (2011). The *Leishmania donovani* UMP synthase is essential for promastigote viability and has an unusual tetrameric structure that exhibits substrate-controlled oligomerization. J. Biol. Chem..

[bib29] Ghosh M., Mukherjee T. (2000). Stage-specific development of a novel adenosine transporter in *Leishmania donovani* amastigotes. Mol. Biochem. Parasitol..

[bib30] Gould M.K., Vu X.L., Seebeck T., De Koning H.P. (2008). Propidium iodide-based methods for monitoring drug action in the kinetoplastidae: comparison with the Alamar Blue assay. Anal. Biochem..

[bib31] Gudin S., Quashie N.B., Candlish D., Al-Salabi M.I., Jarvis S.M., Ranford-Cartwright L.C., De Koning H.P. (2006). *Trypanosoma brucei*: a survey of pyrimidine transport activities. Exp. Parasitol..

[bib32] Hofer A., Ekanem J.T., Thelander L. (1998). Allosteric regulation of *Trypanosoma brucei* ribonucleotide reductase studied in vitro and in vivo. J. Biol. Chem..

[bib33] Katakura K., Fujise H., Takeda K., Kaneko O., Torii M., Suzuki M., Chang K.P., Hashiguchi Y. (2004). Overexpression of LaMDR2, a novel multidrug resistance ATP-binding cassette transporter, causes 5-fluorouracil resistance in *Leishmania amazonensis*. FEBS Lett..

[bib34] LaFon S.W., Nelson D.J., Berens R.L., Marr J.J. (1982). Purine and pyrimidine salvage pathways in *Leishmania donovani*. Biochem. Pharmacol..

[bib35] Landfear S.M. (2001). Molecular genetics of nucleoside transporters in *Leishmania* and African trypanosomes. Biochem. Pharmacol..

[bib36] Liu W., Boitz J.M., Galazka J., Arendt C.S., Carter N.S., Ullman B. (2006). Functional characterization of nucleoside transporter gene replacements in *Leishmania donovani*. Mol. Biochem. Parasitol..

[bib37] Lüscher A., De Koning H.P., Mäser P. (2007). Chemotherapeutic strategies against *Trypanosoma brucei*: drug targets vs. drug targeting. Curr. Pharm. Des..

[bib38] Lüscher A., Lamprea-Burgunder E., Graf F.E., De Koning H.P., Mäser P. (2013). *Trypanosoma brucei* adenine-phosphoribosyltransferases mediate adenine salvage and aminopurinol susceptibility but not adenine toxicity. Int. J. Parasitol. Drugs Drug Resist.

[bib39] Lüscher A., Önal P., Schweingruber A.M., Mäser P. (2007). Adenosine kinase of *Trypanosoma brucei* and its role in susceptibility to adenosine antimetabolites. Antimicrob. Agents Chemother..

[bib40] Matovu E., Stewart M., Geiser F., Brun R., Mäser P., Wallace L.J.M., Burchmore R.J., Enyaru J.C.K., Barrett M.P., Kaminsky R., Seebeck T., De Koning H.P. (2003). The mechanisms of arsenical and diamidine uptake and resistance in *Trypanosoma brucei*. Eukaryot. Cell.

[bib41] Munday J.C., Eze A.A., Baker N., Glover L., Clucas C., Aguinaga Andrés D., Natto M.J., Teka I.A., McDonald J., Lee R.S., Graf F.E., Ludin P., Burchmore R.J.S., Turner C.M.R., Tait A., MacLeod A., Mäser P., Barrett M.P., Horn D., De Koning H.P. (2014). *Trypanosoma brucei* Aquaglyceroporin 2 is a high affinity transporter for pentamidine and melaminophenyl arsenic drugs and is the main genetic determinant of resistance to these drugs. J. Antimicrob. Chemother..

[bib42] Munday J.C., Rojas Lopez K.E., Eze A.A., Delespaux V., Van Den Abbeele J., Rowan T., Barrett M.P., Morrison L.J., De Koning H.P. (2013). Functional expression of TcoAT1 reveals it to be a P1-type nucleoside transporter with no capacity for diminazene uptake. Int. J. Parasitol. Drugs Drug Resist.

[bib43] Munday J.C., Tagoe D.N.A., Eze A.A., Krezdorn J.A., Rojas López K.E., Alkhaldi A.A.M., McDonald F., Still J., Alzahrani K.J., Settimo L., De Koning H.P. (2015). Functional analysis of drug resistance-1 associated mutations in the *Trypanosoma brucei* Adenosine Transporter 1 (TbAT1) and the proposal of a structural model for the protein. Mol. Microbiol..

[bib44] Ortiz D., Sanchez M.A., Koch H.P., Larsson H.P., Landfear S.M. (2009). An acid-activated nucleobase transporter from *Leishmania major*. J. Biol. Chem..

[bib45] Ortiz D., Sanchez M.A., Pierce S., Herrmann T., Kimblin N., Archie Bouwer H.G., Landfear S.M. (2007). Molecular genetic analysis of purine nucleobase transport in *Leishmania major*. Mol. Microbiol..

[bib46] Papageorgiou I.G., Yakob L., Al Salabi M.I., Diallinas G., Soteriadou K.P., De Koning H.P. (2005). Identification of the first pyrimidine nucleobase transporter in *Leishmania*: similarities with the *Trypanosoma brucei* U1 transporter and antileishmanial activity of uracil analogues. Parasitology.

[bib47] Quashie N.B., Dorin-Semblat D., Bray P.G., Biagini G.A., Doerig C., Ranford-Cartwright L.C., De Koning H.P. (2008). A comprehensive model of purine uptake by the malaria parasite *Plasmodium falciparum*: identification of four purine transport activities in intraerythrocytic parasites. Biochem. J..

[bib48] Ranjbarian F., Vodnala M., Alzahrani K.J., Ebiloma G.U., De Koning H.P., Hofer A. (2017). 9-(2-Deoxy-2-fluoro-ß-D-arabinofuranosyl) adenine as a therapeutic agent against *Trypanosoma brucei*. Antimicrob. Agents Chemother..

[bib49] Räz B., Iten M., Grether-Bühler Y., Kaminsky R., Brun R. (1997). The Alamar Blue assay to determine drug sensitivity of African trypanosomes (*T. b. rhodesiense* and *T. b. gambiense*) in vitro. Acta Trop..

[bib50] Ritt J.F., Raymond F., Leprohon P., Légaré D., Corbeil J., Ouellette M. (2013). Gene amplification and point mutations in pyrimidine metabolic genes in 5-fluorouracil resistant *Leishmania infantum*. PLoS Negl. Trop. Dis..

[bib51] Rodrigues J.C., Godinho J.L., de Souza W. (2014). Biology of human pathogenic trypanosomatids: epidemiology, lifecycle and ultrastructure. Subcell. Biochem..

[bib52] Rodenko B., Wanner M.J., Alkhaldi A.A.M., Ebiloma G.U., Barnes R.L., Kaiser M., Brun R., McCulloch R., Koomen G.J., De Koning H.P. (2015). Targeting the parasite's DNA with methyltriazenyl purine analogs is a safe, selective and efficacious antitrypanosomal strategy. Antimicrob. Agents Chemother..

[bib53] Sanchez M.A., Tyron R., Pierce S., Vasudevan G., Landfear S.M. (2004). Functional expression and characterization of a purine nucleobase transporter gene from *Leishmania major*. Mol. Membr. Biol..

[bib54] Stein A., Vaseduvan G., Carter N.S., Ullman B., Landfear S.M., Kavanaugh M.P. (2003). Equilibrative nucleoside transporter family members from *Leishmania donovani* are electrogenic proton symporters. J. Biol. Chem..

[bib55] Sundar S., Chakravarty J. (2013). Leishmaniasis: an update of current pharmacotherapy. Expert Opin. Pharmacother..

[bib56] Teka I.A., Kazibwe A.J.N., El-Sabbagh N., Al-Salabi M.I., Ward C.P., Eze A.A., Munday J.C., Mäser P., Matovu E., Barrett M.P., De Koning H.P. (2011). The diamidine diminazene aceturate is a substrate for the high-affinity pentamidine transporter: implications for the development of high resistance levels in trypanosomes. Mol. Pharmacol..

[bib57] Thiel M., Harder S., Wiese M., Kroemer M., Bruchhaus I. (2008). Involvement of a *Leishmania* thymidine kinase in flagellum formation, promastigote shape and growth as well as virulence. Mol. Biochem. Parasitol..

[bib58] Timm J., Bosch-Navarrette C., Recio E., Nettleship J.E., Rada H., González-Pacanowska D., Wilson K.S. (2015). Structural and kinetic characterization of thymidine kinase from *Leishmania* major. PloS Negl. Trop. Dis..

[bib59] Titus R.G., Gueiros-Filho F.J., de Freitas L.A., Beverley S.M. (1995). Development of a safe live *Leishmania* vaccine line by gene replacement. Proc. Natl. Acad. Sci. U. S. A..

[bib60] Valente M., Vidal A.E., González-Pacanowska D., Müller S., Cerdan R., Radulescu O. (2016). Potential of pyrimidine metabolism for antitrypanosomal; drug discovery. Comprehensive Analysis of Parasite Biology: from Metabolism to Drug Discovery.

[bib61] Vasudevan G., Carter N.S., Drew M.E., Beverley S.M., Sanchez M.A., Seyfang A., Ullman B., Landfear S.M. (1998). Cloning of *Leishmania* nucleoside transporter genes by rescue of a transport-deficient mutant. Proc. Natl. Acad. Sci. U. S. A..

[bib62] Vickers T.J., Beverley S.M. (2011). Folate metabolic pathways in *Leishmania*. Essays Biochem..

[bib63] Vodnala M., Ranjbarian F., Pavlova A., De Koning H.P., Hofer A. (2016). *Trypanosoma brucei* methylthioadenosine phosphorylase protects the parasite from the antitrypanosomal effect of deoxyadenosine: implications for the pharmacology of adenosine antimetabolites. J. Biol. Chem..

[bib64] Vodnala S.K., Lundbäck T., Yeheskieli E., Sjöberg B., Gustavsson A.L., Svensson R., Olivera G., Eze A.A., De Koning H.P., Hammarström L.G.J., Rottenberg M.E. (2013). Structure-activity relationships of synthetic cordycepin analogues as experimental therapeutics for Africa trypanosomiasis. J. Med. Chem..

[bib65] Wallace L.J.M., Candlish D., De Koning H.P. (2002). Different substrate recognition motifs of human and trypanosome nucleobase transporters: selective uptake of purine antimetabolites. J. Biol. Chem..

[bib66] Wilson Z.N., Gilroy C.A., Boitz J.M., Ullman B., Yates P.A. (2012). Genetic dissection of pyrimidine biosynthesis and salvage in *Leishmania donovani*. J. Biol. Chem..

[bib67] Youn J.H., Nam H.W., Kim D.J., Choi W.Y. (1990). Effects of pyrimidine salvage inhibitors on uracil incorporation of *Toxoplasma gondii*. Kisaengchunghak Chapchi.

