# Supplementary material for: Functional and genetic evidence that nucleoside transport is highly conserved in Leishmania species: Implications for pyrimidine-based chemotherapy
Source: Int J Parasitol Drugs Drug Resist. 2017 Apr 20;7(2):206–26. doi: 10.1016/j.ijpddr.2017.04.003 (PMC5407577; doi:10.1016/j.ijpddr.2017.04.003)
Supplement: Online data [file mmc1.docx]

Supplemental Table S1. Nucleoside transporter genes in *L. major* and *L. mexicana*

| Strain | Gene ID | GenBank^TM^ accession number | The author’s designated name |
| --- | --- | --- | --- |
| *L. major* | LmjF15.1230 | XM_001681987 | *LmaNT1A* |
|  | LmjF15.1240 | XM_001681988 | *LmaNT1B* |
|  | LmjF.36.1940 | XM_001686717 | *LmaNT2* |
| *L. mexicana* | LmxM.15.1230 | XM_003873593 | *LmexNT1A* |
|  | LmxM.15.1240 | XM_003873594 | *LmexNT1B* |
|  | LmxM.36.1940 | XM_003874493 | *LmexNT2* |

Supplemental table S2. List of primers used in this study

| Gene | Primer direction | Sequence (5’-3’) | Amplicon size (bp) |
| --- | --- | --- | --- |
| *LmaNT1A* | Forward | GACTAAGCTTATGCCTGCGTGTGTGCC | 1971 |
|  | Reverse | AATTGGATCCTCAGTGGCGCTCGCG |  |
| *LmaNT1B* | Forward | AACCAAGCTTATGGACACCGCATCCGA | 1476 |
|  | Reverse | AATTGGATCCCTAGTATGTCGCGCGGA |  |
| *LmaNT2* | Forward | AATCAAGCTTATGACGGGCCAATCTGC | 1500 |
|  | Reverse | GGCCGGATCCTTAGTAGGTCAGAGTGA |  |
| *LmexNT1A* | Forward | AATTAAGCTTATGGACACCGCGCCCGA | 1476 |
|  | Reverse | AATTGGATCCTCAGTGACGCTCGCGGA |  |
| *LmexNT1B* | Forward | AATTAAGCTTATGGACACCGCGCCCGA | 1476 |
|  | Reverse | GACTGGATCCTTAGTAAGTCGCGCGGA |  |
| *LmexNT2* | Forward | GCTTAAGCTTATGACGGGCCAATCTGC | 1500 |
|  | Reverse | GCGTGGATCCTTAGTAGGTCAGGGTAA |  |

Supplemental table S3: List of primers used in this study for qRT-PCR

| Gene | Forward primer | Reverse primer |
| --- | --- | --- |
| LmaNT1A | TCCGCGCCTGCATACATT | GCTTCCGGGTCCTGCAT |
| LmaNT1B | TCCGCGCCTGCATACATT | GCTTCCGGGTCCTGCAT |
| LmaNT2 | GCCTCCGTGTGGTCTGTGT | GAAGGCGCACAGGAGCAT |
| LmexNT1A | GCTTCTCGCCGTCGTTGA | ATGATCCAGCGCTGCTTGTA |
| LmexNT1B | GCTTCTCGCCGTCGTTGA | ATGATCCAGCGCTGCTTGT |
| LmexNT2 | CGGTGATGGGTGGCTTCT | GCCGACAAGAGCGTTCGT |
| *T. b. b.* GPI8 | TCTGAACCCGCGCACTTC | CCACTCACGGACTGCGTTT |
| *Leishmania* GPI8 | GGCTGTCATTGTCTCCTCCT | GTACATGGTAAGCGCATTGG |

**Supplementary Figure Legends.**

Supplemental Figure S1. The expression of *L. major* NT1A, NT1B and NT2 (A) and *L. mexicana* NT1A, NT1B and NT2 in *T. b. b.* B48 cells compared to B48 control cells. Expression of nucleoside transporter genes was assessed by qRT-PCR. The results are presented normalized to *GPI8* expression levels. Error bars show Average ± SEM. Unpaired Student’s t-test: ^*^ *P*>0.05, ^**^ *P*>0.01, ^***^ *P*>0.001; n=3-6).

Supplemental Figure S2. Gene expression analysis of NT1 and NT2 genes in *L. mexicana* amastigotes and promastigotes. Difference in expression was evaluated by unpaired Student’s t-test (*L. mexicana* amastigotes vs. *L. mexicana* promastigotes) and found to be not significant (*P*>0.05). Error bars represent standard deviation (n = 3).

Supplemental Figure S3. Expression of (A) LmexNT1A, (B) LmexNT1B, (C) LmajNT1A, and (D) LmajNT1B in *T. brucei* strain B48. Transport of 0.5 µM [^3^H]-uridine (●) was measured over 600 s and inhibited with either 1 mM unlabelled uridine (▲) or 250 µM adenosine (□).

Supplemental Figure S4. The sensitivity of *L. mexicana* promastigotes (A) and *L. major* promastigotes (B) to 5-FU (●), 5F-2’dUrd (∆) and 5F-Urd (□), using Alamar Blue assays.

Supplemental Figure S5. Adaptation of promastigotes of *Leishmania spp* to high concentrations of fluorinated pyrimidine analogs during in vitro culturing. Concentrations indicated are the concentrations of analog added to the medium, in which the cells managed to survive and multiply. This was 5-fluorouracil to generate the adapted cell line-5FURes, and 5F-2’dUrd to generate the 5F-dURes cell lines. After adaptation of the cultures the promastigotes were cloned out by limiting dilution so that the eventual cell lines that were characterized were all grown from a single cell.
