# Supplementary material for: Functional and genetic evidence that nucleoside transport is highly conserved in Leishmania species: Implications for pyrimidine-based chemotherapy
Source: Int J Parasitol Drugs Drug Resist. 2017 Apr 20;7(2):206–26. doi: 10.1016/j.ijpddr.2017.04.003 (PMC5407577; doi:10.1016/j.ijpddr.2017.04.003)
Supplement: Supplementary Fig. s1–s5 [file mmc2.pptx]

## Slide 1
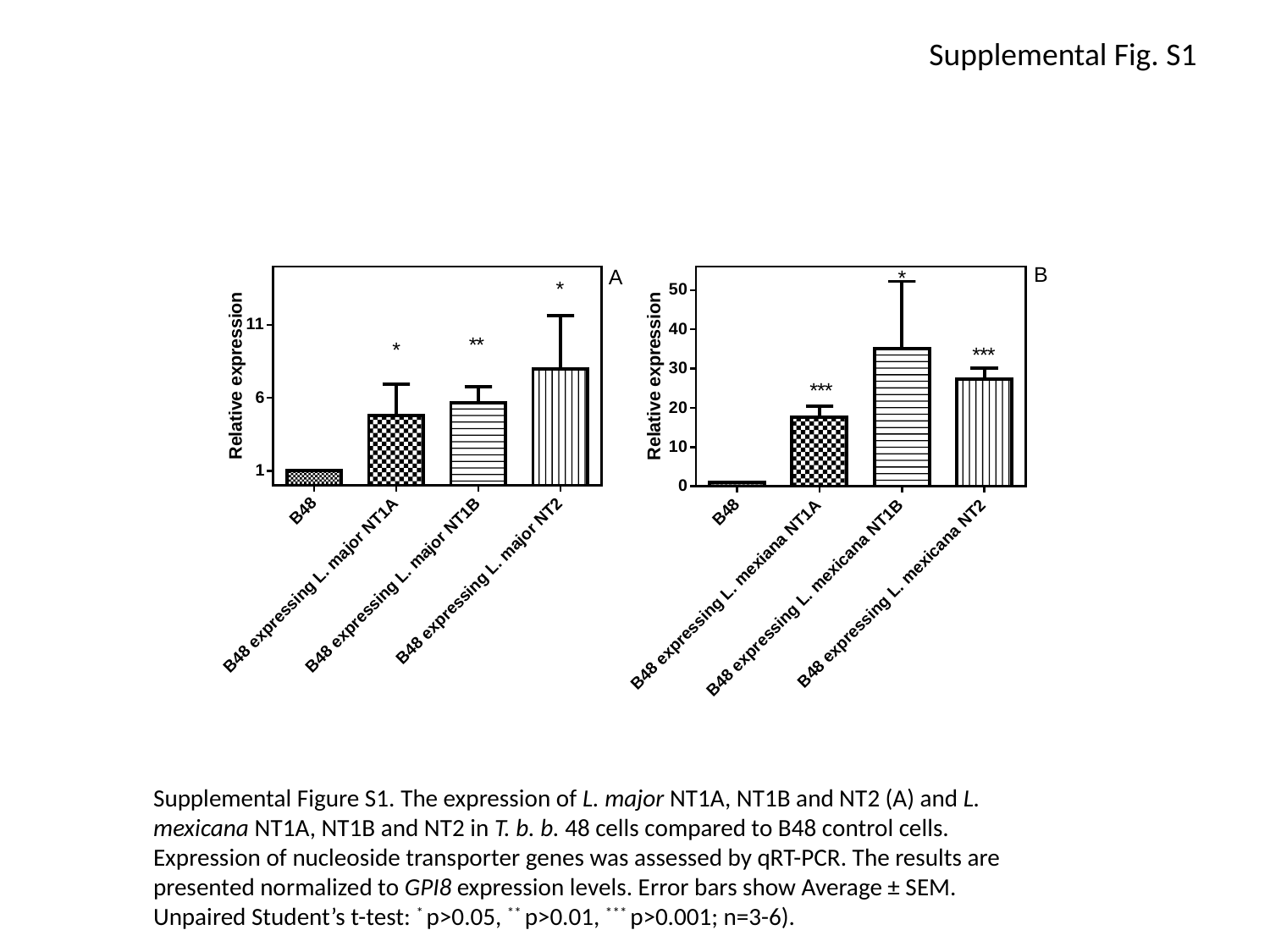

Supplemental Fig. S1
Supplemental Figure S1. The expression of L. major NT1A, NT1B and NT2 (A) and L. mexicana NT1A, NT1B and NT2 in T. b. b. 48 cells compared to B48 control cells. Expression of nucleoside transporter genes was assessed by qRT-PCR. The results are presented normalized to GPI8 expression levels. Error bars show Average ± SEM. Unpaired Student’s t-test: * p>0.05, ** p>0.01, *** p>0.001; n=3-6).

## Slide 2
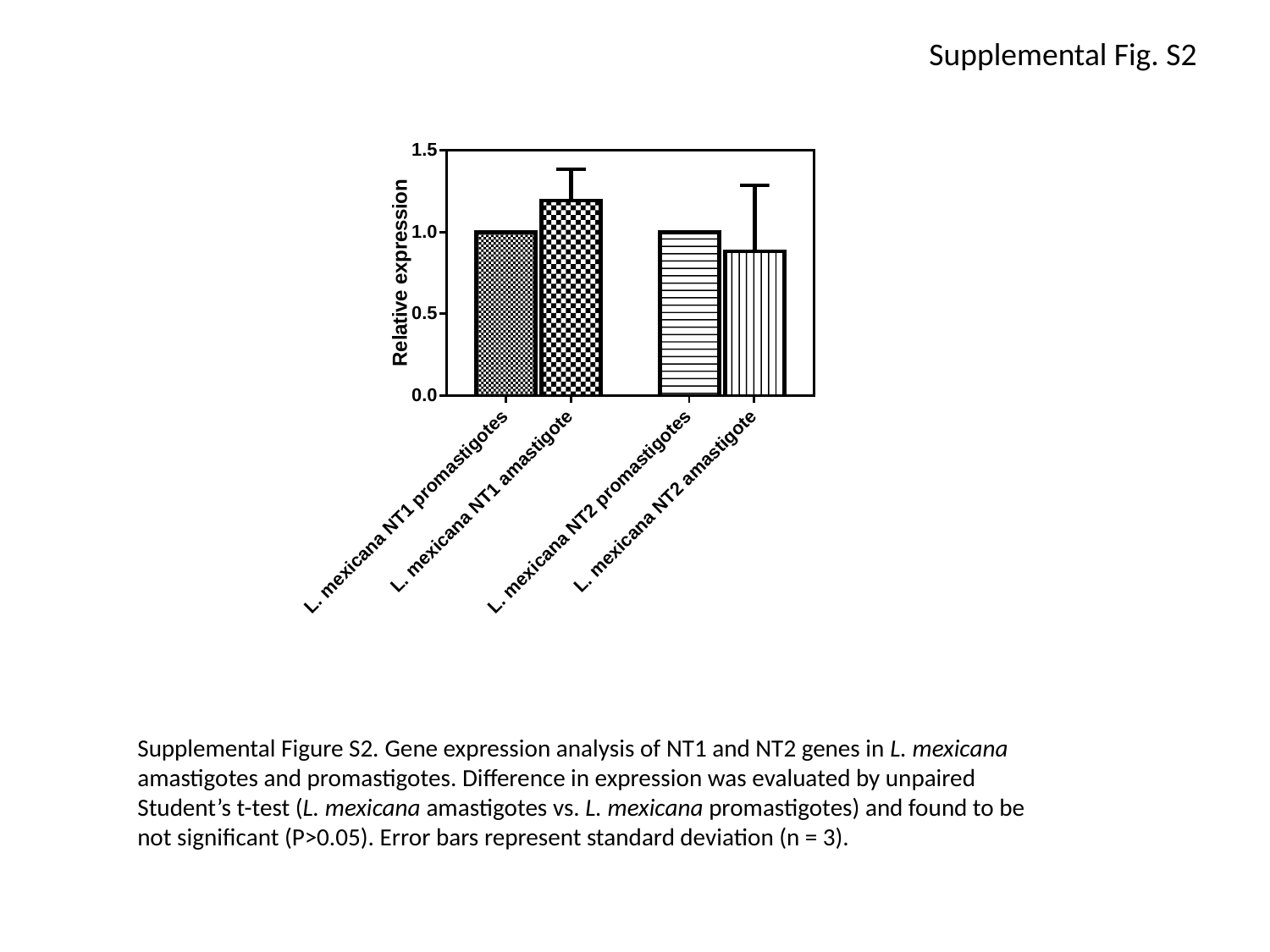

Supplemental Fig. S2
Supplemental Figure S2. Gene expression analysis of NT1 and NT2 genes in L. mexicana amastigotes and promastigotes. Difference in expression was evaluated by unpaired Student’s t-test (L. mexicana amastigotes vs. L. mexicana promastigotes) and found to be not significant (P>0.05). Error bars represent standard deviation (n = 3).

## Slide 3
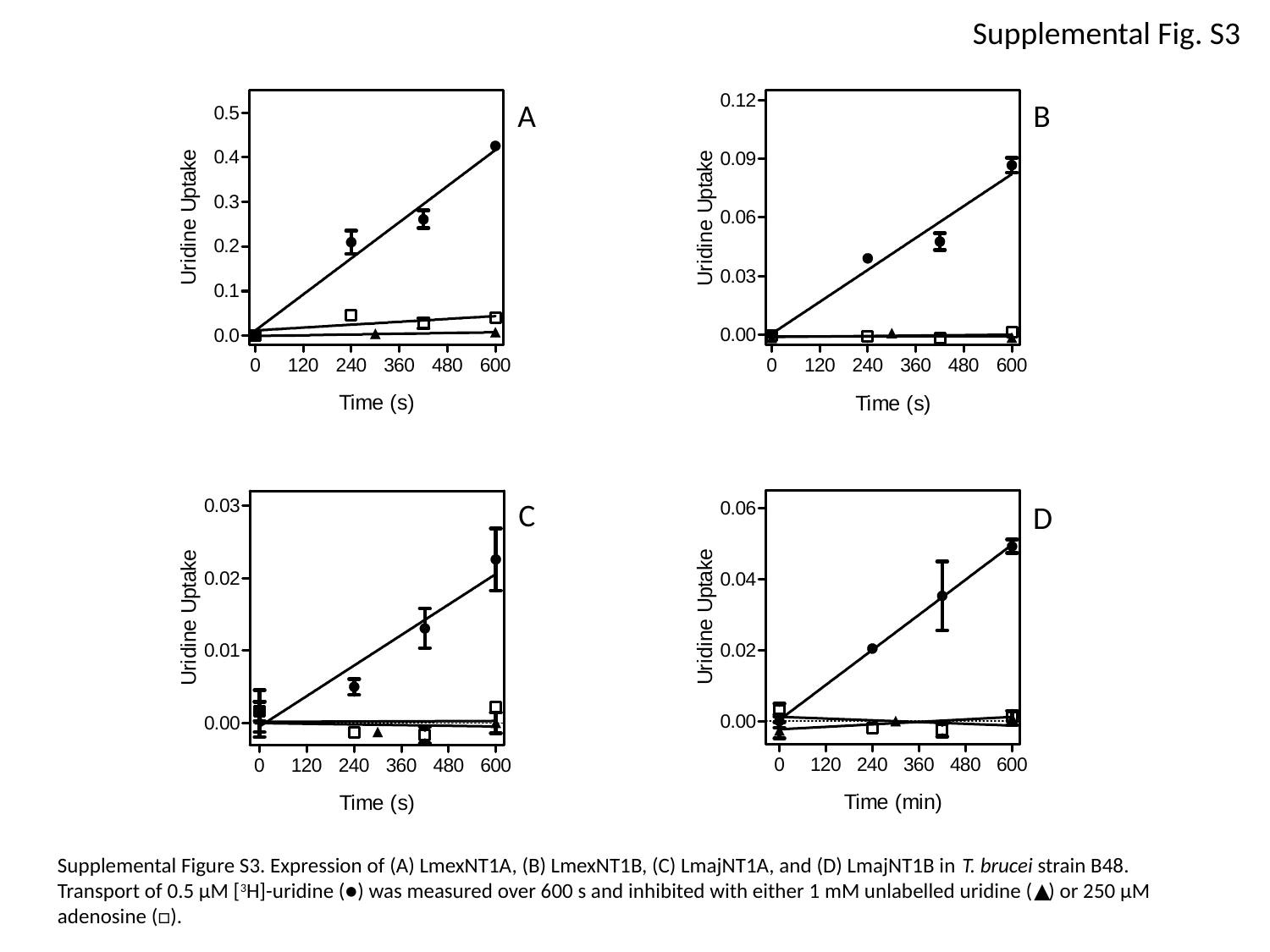

Supplemental Fig. S3
A
B
C
D
Supplemental Figure S3. Expression of (A) LmexNT1A, (B) LmexNT1B, (C) LmajNT1A, and (D) LmajNT1B in T. brucei strain B48. Transport of 0.5 µM [3H]-uridine (●) was measured over 600 s and inhibited with either 1 mM unlabelled uridine (▲) or 250 µM adenosine (□).

## Slide 4
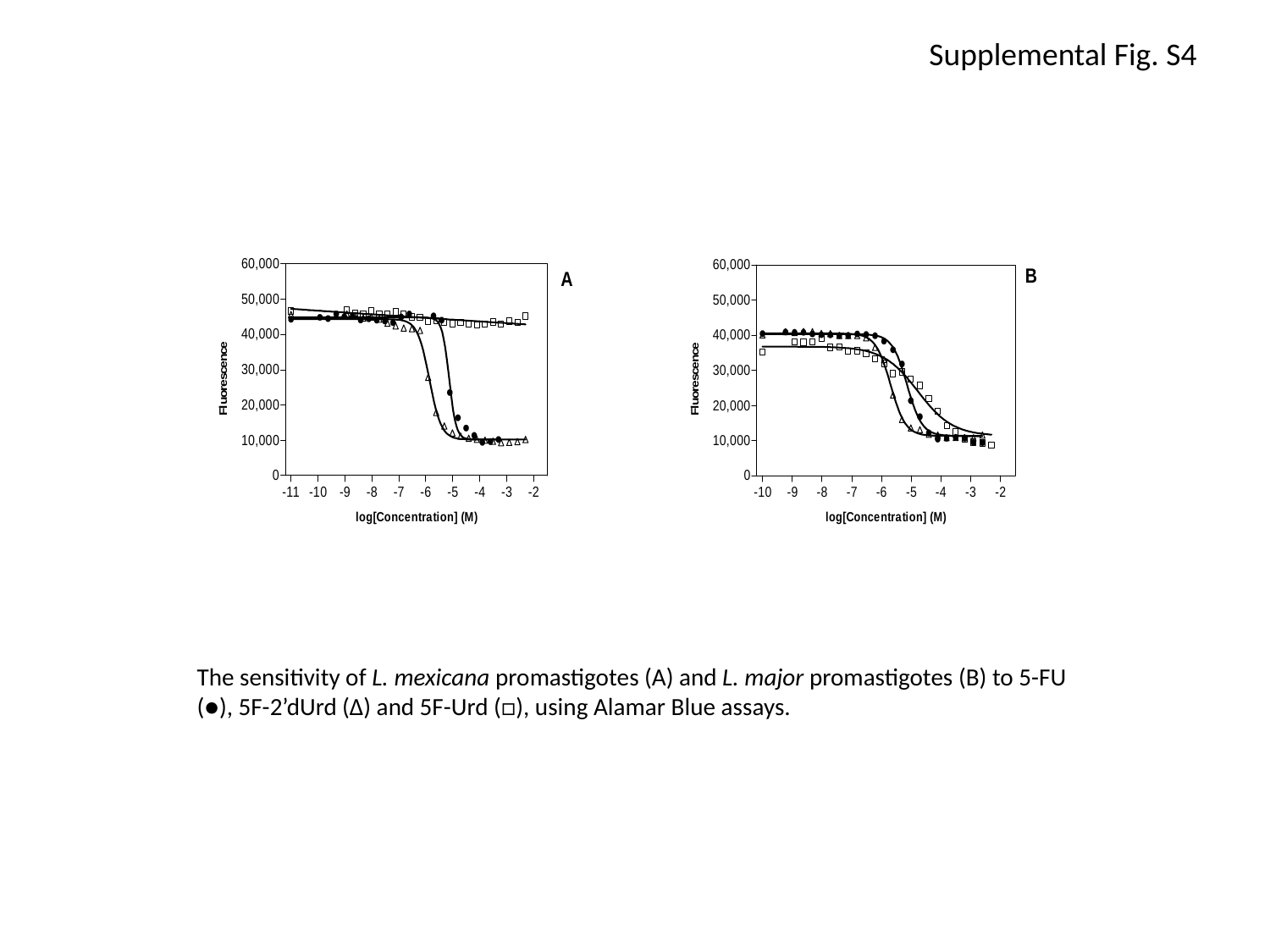

Supplemental Fig. S4
The sensitivity of L. mexicana promastigotes (A) and L. major promastigotes (B) to 5-FU (●), 5F-2’dUrd (∆) and 5F-Urd (□), using Alamar Blue assays.

## Slide 5
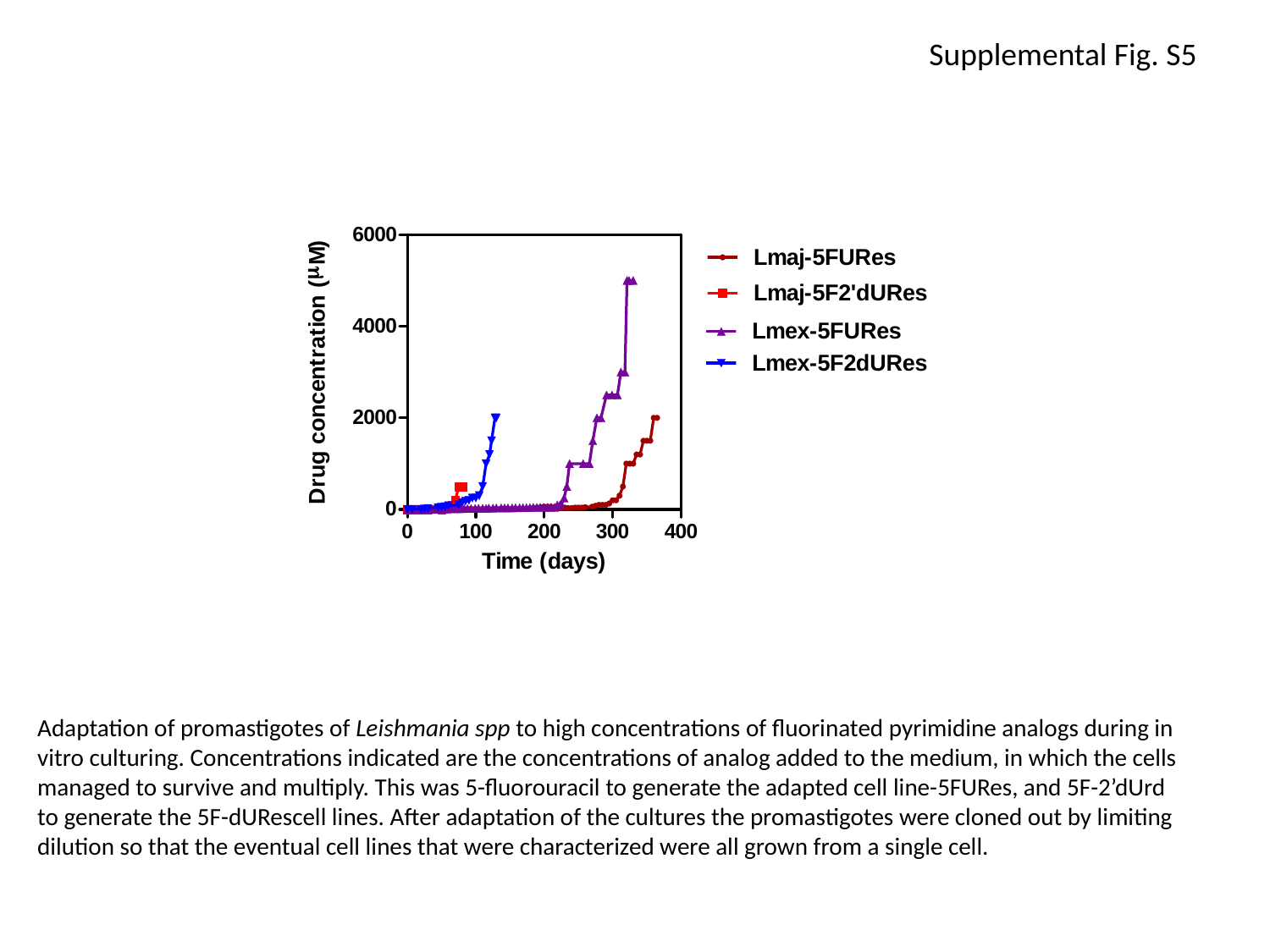

Supplemental Fig. S5
Adaptation of promastigotes of Leishmania spp to high concentrations of fluorinated pyrimidine analogs during in vitro culturing. Concentrations indicated are the concentrations of analog added to the medium, in which the cells managed to survive and multiply. This was 5-fluorouracil to generate the adapted cell line-5FURes, and 5F-2’dUrd to generate the 5F-dURescell lines. After adaptation of the cultures the promastigotes were cloned out by limiting dilution so that the eventual cell lines that were characterized were all grown from a single cell.
